# Excitatory/inhibitory ratio disruption modulates neural synchrony and flow directions in a cortical microcircuit

**DOI:** 10.1371/journal.pcbi.1013306

**Published:** 2025-08-06

**Authors:** Nobuhiko Wagatsuma, Sou Nobukawa, Tomoki Kurikawa

**Affiliations:** 1 Faculty of Science, Toho University, Funabashi, Chiba, Japan; 2 Department of Computer Science, Narashino, Chiba Institute of Technology, Chiba, Japan; 3 Department of Preventive Intervention for Psychiatric Disorders, National Institute of Mental Health, National Center of Neurology and Psychiatry, Kodaira, Tokyo, Japan; 4 Department of Complex and Intelligent Systems, Future University Hakodate, Hakodate, Hokkaido, Japan; Royal Institute of Technology (KTH), SWEDEN

## Abstract

Autism spectrum disorder (ASD) and schizophrenia are complex and heterogeneous mental disorders involving the dysfunction of multiple neural systems. The atypical and heterogenous temporal coordinations of neuronal activity, which are widely observed in these two disorders, are hypothesized to stem from an excitatory/inhibitory (E/I) imbalance in the brain. To investigate the association between the E/I imbalance and atypical neural activities, and to assess the influence of specific subtypes of inhibitory interneurons on network activity regulation, we developed a computational microcircuit model with biologically plausible layer 2/3 of visual cortex that combined excitatory pyramidal neurons with three subtypes of inhibitory interneurons (parvalbumin [PV], somatostatin [SOM], and vasoactive intestinal polypeptide [VIP]). We numerically explored the role of distinct types of E/I imbalance by changing the population size of different subtype neurons. We find that when the E/I balance is disrupted by decreasing the PV population size, activity of the PV population precedes that of the pyramidal population, which enhances beta and gamma oscillations. Conversely, pyramidal neuronal population activity was the precursor of PV interneuron activity when the E/I imbalance was induced by decreasing the SOM population size; this preferentially impaired gamma-frequency activity. The disruption of E/I balance altered the information flow between pyramidal and PV populations, modulating neuronal dynamics. Our results suggest that E/I imbalance due to different subtype interneurons would induce the distinct types of the atypical neural behaviors associated with neural system dysfunction.

## Author summary

Autism spectrum disorder and schizophrenia involve complex neural system dysfunction, often linked to various types of excitatory/inhibitory (E/I) imbalances in the brain. To elucidate how these imbalances influence neural activity, we developed a computational model of layer 2/3 of the visual cortex, comprising excitatory pyramidal neurons and three subtypes of inhibitory interneurons: parvalbumin (PV), somatostatin (SOM), and vasoactive intestinal polypeptide (VIP). We numerically explored the roles of distinct types of E/I imbalance by changing the population size of different subtype neurons. A reduction in PV population enhanced beta and gamma oscillations, with changes in PV activity preceding those in pyramidal neurons. Conversely, reducing SOM population selectively impaired gamma oscillations, with pyramidal neuron activity preceding changes in PV interneuron activity. These results suggest that E/I imbalances arising from different interneuron subtypes produce distinct neural activity patterns, potentially underlying the atypical neural behaviors associated with neural system dysfunction.

## Introduction

The superficial layers (2 and 3) of the primary visual area (V1) are a crucial neural circuit for processing visual information and establishing perception of the external world [1]. In the V1, the fundamental components of this cortical microcircuit are excitatory pyramidal (Pyr) neurons and three distinct subtypes of inhibitory interneurons, classified based on the expression of one of three genes: parvalbumin (PV), somatostatin (SOM), or vasoactive intestinal polypeptide (VIP) [2–6]. Interactions among these neuronal populations are essential for integrating various signals, including feedforward visual inputs from layer 4 of the primary input station in the layered network and horizontal connections and for generating synchronized responses [7–10].

Autism spectrum disorder (ASD) and schizophrenia (SZ) are a group of complex and heterogeneous mental disorders that involve the dysfunction of multiple neural systems. Patients with ASD and SZ often have not only impairments in visual perception [11–13], but also impaired temporal coordination of neuronal activity in cortical circuits [14–17]. These impaired neuronal dynamics and the perceptual alternations noted in these disorders are hypothesized to stem from an excitatory/inhibitory (E/I) imbalance in the brain. Specifically, previous studies have reported that a shift toward heightened excitability and diminished inhibition in the E/I balance underlies the pathophysiology of SZ and ASD [18–28].

Physiologically detailed circuit models are essential for revealing the mechanisms of perception through the complex interactions between excitatory and inhibitory classes or various neuronal subtypes [29–34]. For example, Nobukawa et al. [35] has investigated computationally the influences of E/I balance disruptions on change in the neural oscillations by altering the numbers of excitatory and inhibitory neurons in their model. However, this network consisted of just two neuronal classes: excitatory and inhibitory. The effects of E/I balance disruption caused by altering the population size of specific interneuron subtypes have not yet been explored. Given that different subtypes of inhibitory neurons play distinct roles in regulating and modulating neuronal oscillations [7,8], their involvement in pathological changes in cortical circuits, such as impaired temporal coordination of neuronal activity in complex mental disorders, remains to be elucidated. In particular, the impact of E/I imbalance resulting from variations in the population size of specific interneuron subtypes within the functional microcircuit may provide crucial insights into the relationship between atypical neural activity and neural system dysfunction within fundamental brain networks.

**Fig 1:**
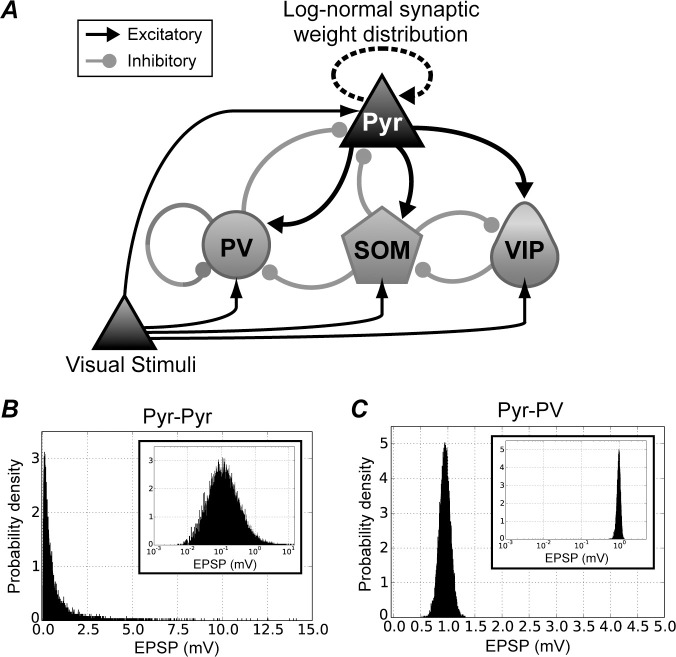
Architecture of the proposed microcircuit model for layers 2/3 of the V1. ***A.*** The microcircuit model was composed of four neuronal populations: one excitatory pyramidal (Pyr) neuron population and three inhibitory interneuron subtype (parvalbumin [PV], somatostatin [SOM], and vasoactive intestinal polypeptide [VIP]) populations. The Pyr, PV, SOM, and VIP populations are illustrated by triangle, circle, pentagon, and teardrop shapes, respectively. These neurons in the microcircuit model were described by integrate-and-fire model neurons. Excitatory and inhibitory synaptic connections are represented by arrows with triangular and circular heads, respectively. Excitatory synaptic connections in the microcircuit network, including feedforward inputs (visual stimuli), were provided by AMPA receptors. Synaptic strengths between excitatory Pyr neurons obeyed a log-normal distribution [36–39]. Synaptic strengths for excitatory-to-inhibitory, inhibitory-to-excitatory, and inhibitory-to-inhibitory connections obeyed a Gaussian distribution. ***B.*** Distribution of synaptic strengths for excitatory-to-excitatory connections. The strengths of connections between Pyr neurons were determined according to a log-normal amplitude distribution of excitatory postsynaptic potentials. Note that the *x*-axis of the inset graph is shown as a log scale. The main plot is a normal plot of the same distribution. ***C.*** Example distribution of synaptic strengths for excitatory-to-inhibitory neurons (from Pyr to PV neuron populations). All synaptic strengths except for excitatory-to-excitatory connections obeyed a Gaussian distribution.

In the present study, to investigate the associations between the E/I imbalance and the atypical neural behaviors and to assess the influence of specific subtypes of inhibitory interneurons on network activity regulation, we developed a microcircuit model with biologically plausible layers 2/3 in visual cortex, combining excitatory Pyr neurons and three inhibitory interneuron subtypes (PV, SOM, and VIP) (Fig 1). We then numerically explored the effect of different types of E/I imbalance by changing population size of different subtypes of interneurons. Our results show that, when the E/I ratio is enhanced by reducing the PV population size, activity in the PV population precede that in the Pyr. The rapid activation and decay of the PV population lead to enhanced neuronal firing of the microcircuit at beta and gamma frequencies. By contrast, Pyr neuronal activity is the precursor to PV activity when the E/I ratio is increased by decreasing the SOM neuronal population; this preferentially impairs neuronal activity of the microcircuit at gamma frequency. Analyses of our model responses suggest that PV and SOM inhibitory interneurons differentially affect atypical temporal coordination of neuronal activity in the cortical circuits caused by E/I imbalance. Furthermore, disruptions in the E/I ratio caused by different interneuron subtypes significantly modulate the directional flow between Pyr and PV populations in distinct ways. If alterations in the E/I ratio driven by changes in the population sizes of Pyr neurons and specific inhibitory interneuron subtypes within the microcircuit represent a common structural feature of the V1 or other areas in patients with ASD and SZ, then modulation of information flow between Pyr and PV populations may be a widespread phenomenon in these regions. The simulation results from our microcircuit model might provide important insights into the atypical neural structures and behaviors associated with neural system dysfunction arising from E/I imbalances.

## Results

**Fig 2:**
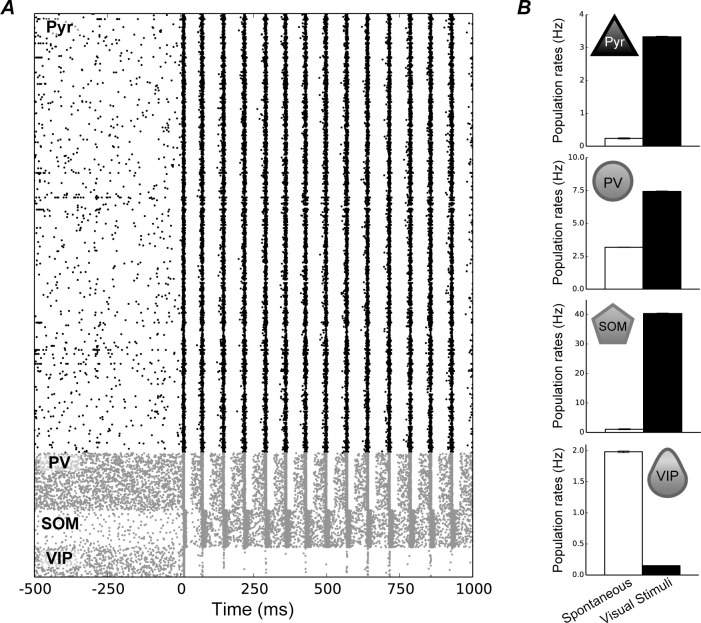
Neuronal responses in the proposed microcircuit model with a ratio of excitatory to inhibitory neurons of 3.5:1.0. ***A.*** Raster plots showing all spike trains of the pyramidal (Pyr), parvalbumin (PV), somatostatin (SOM), and vasoactive intestinal polypeptide (VIP) neuron populations for 1,500 ms. Spikes of excitatory Pyr neurons and the three subtypes of inhibitory interneurons are illustrated by black and gray dots, respectively. For this plot, visual stimuli were given from 0 ms in this panel. ***B.*** The mean population firing rates of Pyr, PV, SOM, and VIP neurons in the microcircuit model from 50 simulation trials of spontaneous (white) and visual stimuli (black) conditions. Error bars indicate standard errors, which were relatively small in these simulations.

Our microcircuit model represents typical connectivity among different classes and subtypes of neurons in layer 2/3 in the V1 of a functional unit for orientation selectivity; it includes excitatory Pyr neurons and three inhibitory interneuron subtypes (PV, SOM, and VIP). The model comprised around 13,000 integrate-and-fire model neurons (Fig 1) [2–4,30,31], which were divided into 10,341 excitatory Pyr neurons and 2,916 inhibitory interneurons based on previous computational studies [29,33,34,40,41]. The ratio of the numbers of excitatory and inhibitory neurons for this model was approximately 3.5:1.0. In the model, there were 1,341 PV, 875 SOM, and 700 VIP neurons in the inhibitory population. The neural behaviors under the E/I ratio of 3.5:1.0 are summarized in Fig 2, which are used as control behavior in this study. Fig 2A shows the spike raster plots of all neuron classes and subtypes over 1,500 ms (spontaneous activity from -500–0 ms and that with a simulated visual stimulus from 0 to 1000 ms). The population of Pyr model neurons exhibits prominent oscillatory responses when feedforward inputs modeled as Poisson spike trains at 25 Hz are applied (Fig 2A). Although the stimulus is applied into all classes of neurons, it activates Pyr, PV, and SOM (but not VIP) neuronal populations (Fig 2B). Further, it generated oscillatory responses for these activated populations.

To investigate the role of each interneuron subtype in impairing the temporal coordination of neuronal activity underlying pathological abnormalities, we varied E/I ratios by changing the population size of different subtype of interneuron with keeping the total number of neurons. Details of the E/I ratios and the numbers of model neurons used in this study are summarized in Table 1. Here, we defined the E/I ratio as the ratio of the population size of Pyr excitatory neurons to the combined population size of all subtypes of inhibitory interneurons [35]. In the following simulations, visual stimuli were applied to the model. The connection probability between excitatory and inhibitory neuron classes and the total number of neurons in the network were constant across all conditions.

**Table 1:** Number of model neurons in the microcircuit model.

	Neuron #			
E/I ratio	Pyr	PV	SOM	VIP
**3.5: 1.0** **(Control)**	10341	1341	875	700
**3.0: 1.0**	**9943**	**1739**	875	700
**4.0: 1.0**	**10606**	**1076**	875	700
**4.5: 1.0**	**10846**	**836**	875	700
**3.0: 1.0**	**9943**	1341	**1237**	700
**4.0: 1.0**	**10606**	1341	**723**	700
**4.5: 1.0**	**10846**	1341	**370**	700

We performed simulations of the model with different E/I ratios caused by changing the numbers of Pyr neurons and PV or SOM interneurons. Note that the total number of model neurons in the microcircuit remained consistent, irrespective of E/I ratio. Abbreviations: E/I, excitatory/inhibitory; PV, parvalbumin; Pyr, pyramidal; SOM, somatostatin; VIP, vasoactive intestinal polypeptide.

### Disruption of E/I balance by changing PV population size

**Fig 3:**
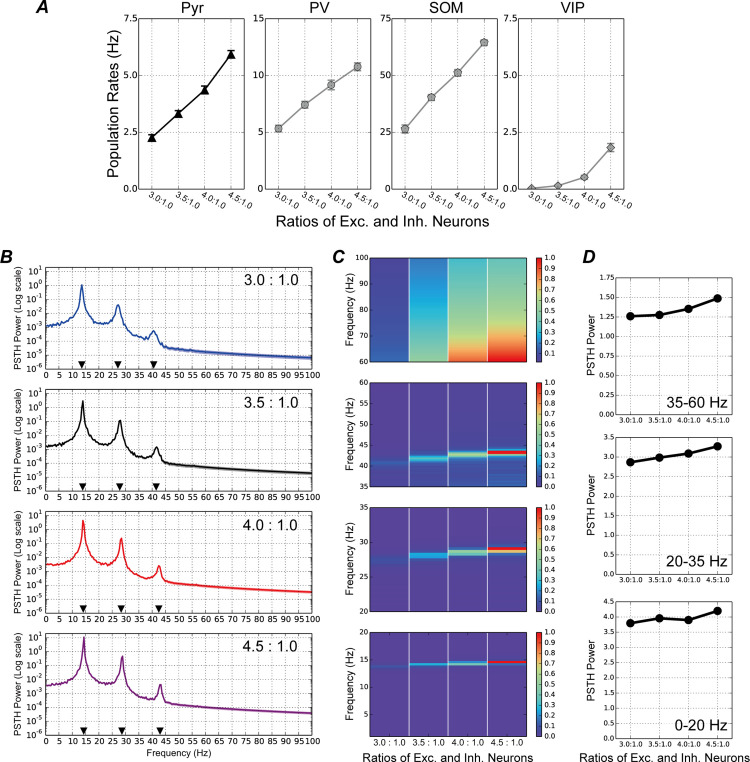
Neuronal responses to visual stimuli in the proposed microcircuit model with different excitatory/inhibitory (E/I) ratios caused by a change in parvalbumin (PV) inhibitory interneuron and pyramidal (Pyr) neuron numbers. Note that the total number of model neurons in the network remained consistent, irrespective of E/I ratio. ***A.*** Mean population firing rates of Pyr (black triangles in first panel from the left), PV (gray circles in the second panel), somatostatin (SOM; gray pentagons in the third panel), and vasoactive intestinal polypeptide (VIP; gray diamonds in the fourth panel) populations as a function of E/I ratios. Error bars represent the standard errors, which were relatively small in these simulations. ***B.*** Peristimulus time histogram (PSTH) power of the Pyr neuron population, averaged over 50 simulation trials with an E/I ratio of 3.0:1.0 (blue line in the first panel from the top), 3.5:1.0 (black line in the second panel), 4.0:1.0 (red line in the third panel), and 4.5:1.0 (purple line in the fourth panel). Note that the *y*-axis is on a log scale. Black triangles indicate the frequencies of the peak in the PSTH power. ***C.*** Normalized PSTH power of the Pyr neuron population across different E/I ratios (normalized within the range of 0–1) for frequency bands ranging 0–20 Hz (the fourth panel from the top), 20–35 Hz (third panel), 35–60 Hz (second panel), and 60–100 Hz (first panel). Color bars illustrate the levels of normalized power strength. Increasing the E/I ratio by increasing Pyr neuron numbers and decreasing PV interneuron numbers markedly activated neuronal activity in both beta and gamma bands for the cortical microcircuit. ***D.*** Relative strengths of the PSTH power peaks at different E/I ratios for frequencies ranging 0–20 Hz (bottom panel), 20–35 Hz (center panel), and 35–65 Hz (top panel). These relative strengths were calculated using the method proposed in reference [42].

Fig 3 summarizes the results for E/I ratios of 3.0:1.0, 3.5:1.0, 4.0:1.0, and 4.5:1.0 caused by changing the size of Pyr and PV populations. We show the spike raster plots of these models with E/I ratios of 3.0:1.0, 4.0:1.0, and 4.5:1.0 in S1, S2 and S3 Figs, respectively. Notably, the E/I ratios were increased here by increasing the number of Pyr neurons and decreasing the number of PV interneurons while maintaining a constant total neuron count. Activities in Pyr neuron and all subtypes of interneuron populations were activated with increasing E/I ratios (Fig 3A).

To analyze characteristics of the temporal coordination of neuronal activity, peristimulus time histograms (PSTHs) of the Pyr population were computed using time bins of 2 ms; these were then decomposed into frequency components using fast Fourier transform (see Materials and Methods). Fig 3B shows the PSTH power of the model’s responses to visual stimuli under various E/I ratios by changing Pyr and PV population sizes. Irrespective of E/I ratios, there were three marked peaks in the PSTH power for alpha (10–20 Hz), beta (20–30 Hz), and low-gamma (30–50 Hz) frequencies, potentially reflecting strongly oscillatory neuronal responses. In addition, PSTH power at the alpha frequency was markedly higher than at lower frequencies, such as around 5 Hz. This pattern may resemble the elevated gamma-frequency power relative to lower frequencies observed in the mouse visual cortex using electroencephalography, at least in part [43]. However, the trends observed in our model simulations appeared more pronounced than those in the biological data.

To analyze further the influence of different E/I ratios on the temporal coordination of neuronal activity, we normalized the PSTH powers shown in Fig 3B (normalized within the range 0–1) across different E/I ratios for specific frequency bands: 10–20 Hz (alpha), 20–35 Hz (beta to low-gamma), 35–60 Hz (low-gamma), and 60–100 Hz (high-gamma) (summarized in Fig 3C). For these frequency ranges, the magnitudes of the peaks of PSTH power were markedly increased with increasing E/I ratios. In particular, increasing E/I ratios from 3.5:1.0 to 4.0:1.0 or 4.5:1.0 elevated the magnitudes of PSTH power for whole ranges of high-gamma band activity (> 60 Hz), whereas decreasing the E/I ratio to 3.0:1.0 suppressed the power of this frequency range. Furthermore, the peaks in the beta (20–35 Hz) and the low-gamma (35–60 Hz) frequencies shifted slightly toward higher frequencies with increasing E/I ratios.

To analyze the PSTH power of the model in more detail under various E/I ratios by changing Pyr and PV population sizes, we extracted the relative strength at three periodic peak locations by removing aperiodic part using a method proposed by Donoghue et al. [42]. Fig 3D shows the relative strength of the PSTH power at the peaks in different E/I ratios for frequencies ranging 0–20 Hz, 20–35 Hz, and 35–60 Hz. The relative strengths of the peaks of PSTH power tended to increase with increasing E/I ratios. These results imply that E/I balance disruption arising from a change in Pyr neuron and PV interneuron numbers markedly modulates neuronal activity in the beta and gamma bands in the cortical microcircuit.

### Disruption of E/I balance by changing SOM population sizes

**Fig 4:**
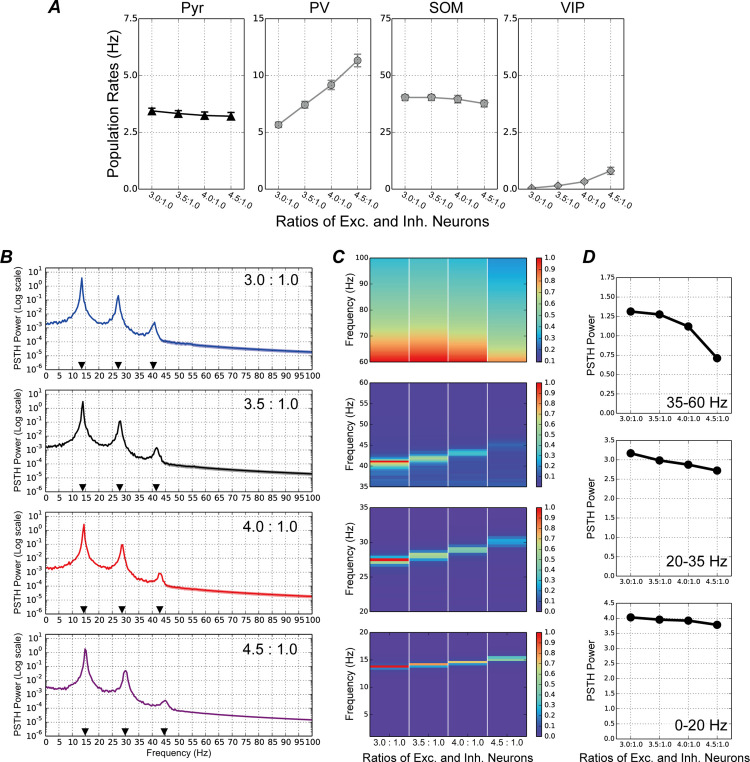
Neuronal responses to visual stimuli in the proposed microcircuit model with different excitatory/inhibitory (E/I) ratios caused by a change in pyramidal (Pyr) neuron and somatostatin (SOM) interneuron numbers. As in Fig 3, the total number of model neurons in the network remained consistent, irrespective of E/I ratio. The conventions of this figure are the same as those of Fig 3. ***A.*** Mean population firing rates of Pyr (first panel from the left), parvalbumin (PV; second panel), SOM (third panel), and vasoactive intestinal polypeptide (VIP; fourth panel) populations as a function of E/I ratios. Error bars represent the standard errors. ***B.*** Peristimulus time histogram (PSTH) powers of the Pyr neuron population, averaged over 50 simulation trials, with an E/I ratio of 3.0:1.0 (the first panel from the top), 3.5:1.0 (second panel), 4.0:1.0 (third panel), and 4.5:1.0 (fourth panel). These plots are shown with a log-scaled *y*-axis. Black triangles indicate frequencies of the peak in the PSTH power. ***C.*** Normalized PSTH power of the Pyr neuron population across various E/I ratios (normalized to within the range of 0–1) for frequency bands ranging 0–20 Hz (fourth panel from the top), 20–35 Hz (third panel), 35–60 Hz (second panel), and 60–100 Hz (first panel). The maximum value in each panel is shown in red. Increasing the E/I ratio by increasing Pyr neuron numbers and decreasing SOM interneuron numbers not only markedly suppressed fast-oscillatory activity in the cortical microcircuit, but also shifted the peak of oscillations toward higher frequencies. ***D.*** The relative strengths of peaks of the PSTH power at various E/I ratios for frequencies ranging 0–20 Hz (bottom panel), 20–35 Hz (center panel), and 35–65 Hz (top panel). These relative strengths were calculated using the method described in reference [42].

To investigate the influence of SOM interneurons on pathological abnormalities, we explored the neural behaviors with various E/I ratios of 3.0:1.0, 3.5:1.0, 4.0:1.0, and 4.5:1.0 by altering the Pyr and SOM population sizes; the responses of the model are shown in Figs 4, S4, S5 and S6. Both Pyr and SOM population activities were slightly suppressed with increasing E/I ratios, whereas other populations were markedly activated (Fig 4A). This finding was in contrast with the modulation of model activity caused by altered PV population sizes (Fig 3A).

We further analyzed the temporal coordination of neuronal activity. The characteristics of synchronized oscillations for various E/I ratios are summarized in Fig 4B. Additionally, as shown in Fig 3C, we normalized the PSTH powers to within the range of 0–1 across various E/I ratios for three specific frequency bands in Fig 4C. In contrast to changing PV population sizes (Fig 3), the magnitudes of the peaks of PSTH power in the three frequency ranges were decreased with increasing E/I ratio. In particular, low-gamma (30–50 Hz) and high-gamma (> 60 Hz) band activity was radically suppressed with an E/I ratio of 4.5:1.0. In addition, increasing E/I ratios markedly shifted the peak locations toward higher frequencies for beta and low-gamma oscillations. Furthermore, analyses of the relative strength at three peak locations [42] for the PSTH power with various E/I ratios by altering the Pyr and SOM population sizes also indicated that the magnitudes of the PSTH power peaks for frequencies ranging 0–20 Hz, 20–35 Hz, and 35–60 Hz decreased with increasing E/I ratios (Fig 4D). These results suggest that increase in the E/I balance by size change of SOM interneuron population not only induced synchronized activity with peaks at higher frequencies, but also reduced the strength of these oscillations. This was distinct from the modulations in the PSTH power induced by PV population change.

### A neuronal-subtype-dependent change in E/I ratios modulates the direction of flows between Pyr and PV populations

So far, these results suggest that different manner of the E/I ratio change by reducing of PV or SOM populations shows contrasting patterns of modulation on neuronal firing at beta and gamma frequencies (Figs 3 and 4). To deepen our understanding of the mechanisms underlying such modulations, we analyzed the direction of flows between Pyr and two subtypes of inhibitory interneuron (PV and SOM) populations using a directed phase lag index (dPLI) [44] for specific frequency ranges (see Materials and Methods). dPLI > 0.5 indicates that the responses in the specific subtype of the inhibitory interneuron population precede those in Pyr population for these frequency ranges. Conversely, dPLI < 0.5 means that the Pyr population responses precede those of the inhibitory interneuron population.

**Fig 5:**
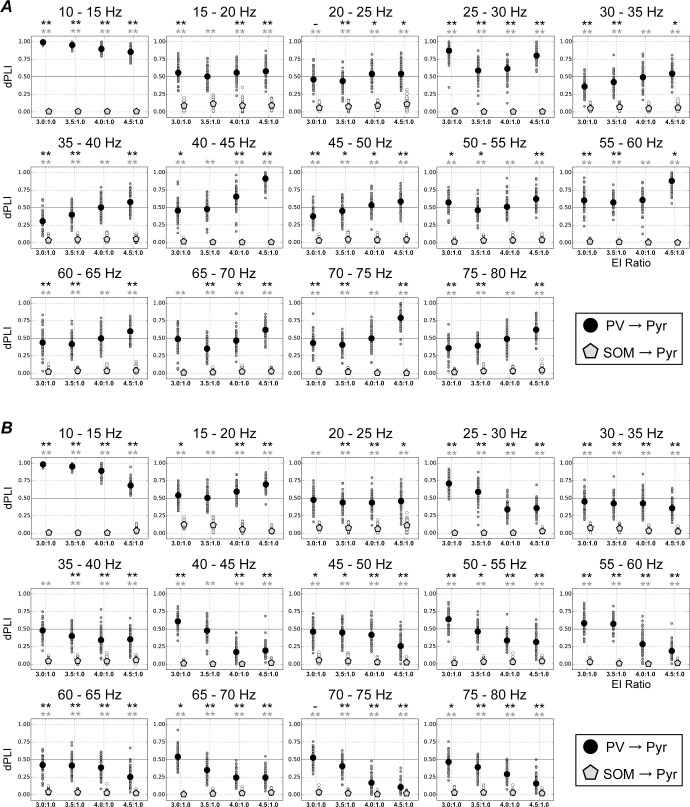
Analyses of directed phase lag index (dPLI [44]) for excitatory/inhibitory (E/I) ratios with respect to pyramidal (Pyr) and parvalbumin (PV) populations (*A*) and Pyr and somatostatin (SOM) populations (*B*) for frequency bands from 10 to 80 Hz (in steps of 5 Hz). Black circles and gray pentagons represent the mean values of dPLI from PV to Pyr and from SOM to Pyr populations, respectively. Values of dPLI from PV to Pyr and from SOM to Pyr populations for each trial are shown by small black and gray circles, respectively. ***A.*** Values of dPLI from PV or SOM inhibitory interneurons to Pyr neurons as a function of E/I ratio with respect to Pyr and PV populations. The dPLI values between SOM and Pyr populations (gray pentagons) were almost consistent, irrespective of frequency band and E/I ratio, whereas increasing the E/I ratio by decreasing the number of PV interneurons increased dPLI values from PV to Pyr populations (black circles) in the range of fast-oscillatory activity. ***B.*** Values of dPLI from PV and SOM to Pyr populations as a function of E/I ratios with respect to Pyr and SOM populations. As shown in the E/I ratio for the PV population (panels *A*), dPLIs from SOM to Pyr populations (gray pentagons) did not fluctuate around values of zero. However, in contrast to the E/I ratio for the PV population, increasing the E/I ratio by decreasing SOM interneuron numbers tended to decrease the dPLI values from PV to Pyr populations. A change in E/I ratios caused by changing between specific subtypes of inhibitory and Pyr populations modulated the dominant direction of flows between PV and Pyr populations. Black and gray asterisks indicate significant differences in the dPLI values from PV to Pyr and SOM to Pyr populations from 0.5, respectively (*t* test, **p < 0.01, *p < 0.05, − *p* < 0.1).

We summarized dPLI values from PV and SOM to Pyr populations for frequency ranges from 10 to 80 Hz in steps of 5 Hz (Fig 5A), as a function of E/I ratio caused by altering Pyr and PV population sizes. Irrespective of the frequency ranges and E/I ratios, mean value levels of dPLI from SOM to Pyr (gray diamonds in Fig 5A) were almost constant at approximately zero, implying that Pyr population responses led SOM responses in these conditions. By contrast, for beta (20–25 Hz), low-gamma (30–60 Hz), and high-gamma (> 60 Hz) band activities, the mean dPLI value levels between Pyr and PV (black circles in Fig 5A) rise with the increase in E/I ratio by reducing the PV population. In particular, with an E/I ratio of 4.5:1.0, mean dPLI value levels between Pyr and PV populations were > 0.5 for firing at beta, low-gamma, and high-gamma band activity. This finding indicates that the activity of PV interneurons precedes that of Pyr neurons in these frequency ranges. However, for the lower E/I ratio (3.0:1.0), mean dPLI value levels were < 0.5 for firing at high-gamma band activity. These results suggest that an E/I imbalance arising from a change in Pyr and PV population sizes might modulate or switch the direction of flows between Pyr and PV populations.

We also applied dPLI analyses to the responses of our model to various E/I ratios caused by changing Pyr neuron and SOM interneuron population sizes. Fig 5B shows the resulting dPLI values between PV or SOM and Pyr populations for frequency ranges from 10 to 80 Hz in steps of 5 Hz, as a function of E/I ratios. In contrast to the dPLI analyses of E/I ratios arising from a change in Pyr and PV population sizes (Fig 5A) for beta- (20–30 Hz), low-gamma (30–60 Hz), and high-gamma (> 60 Hz) band activity, an increase in the E/I ratio by decreasing the SOM population tended to decrease dPLI values. In addition, for these frequency ranges, increasing the E/I ratio to 4.5:1.0 induced dPLI values of < 0.5 between Pyr and PV populations (black circles in Fig 5B). This finding indicates that Pyr neuronal activity is a precursor of PV activity under this condition. However, mean dPLI values between Pyr and SOM populations were almost constant, at approximately zero, for all frequency bands and E/I ratios, similar to the model with different E/I ratios caused by changing Pyr and PV population sizes (Fig 5A). These results suggest that increasing the E/I ratio by increasing Pyr and decreasing SOM population sizes facilitates neural transmission from Pyr neurons to PV interneurons.

Additionally, S7 Fig summarizes the dPLI values across frequency ranges from 10 to 80 Hz in broader 10 Hz steps, for various E/I ratios resulting from changes in the population sizes of Pyr neurons and either PV or SOM interneurons. dPLI values computed with broader frequency steps exhibit modulation patterns similar to those observed with 5 Hz steps, indicating consistent effects of E/I ratio disruptions (Fig 5). Intriguingly, the modulation patterns of dPLI values associated with the PSTH power peaks in the 10–20 Hz, 20–30 Hz, and 40–50 Hz frequency bands under the synchronous conditions (Figs 3 and 4) resemble those observed in other frequency ranges under more asynchronous conditions. These results suggest that although harmonics in the model responses may partially influence the dPLI values, the modulation of information flow direction induced by disruptions in the E/I ratio may still be preserved under more asynchronous conditions.

Together, our dPLI analyses show that E/I ratio disruptions due to different types of interneuron markedly modulate the direction of flows between Pyr and PV populations in distinctive manner; this might then cause the different modulations of neuronal firing at beta and gamma frequencies. The detailed mechanisms underlying these modulations will be discussed further in the Discussion. Additionally, the relationship between the dPLI analysis and oscillatory neuronal responses, particularly in relation to the direction of information flow within the network, will also be discussed in the Discussion section.

We also calculated the phase lag index (PLI) between Pyr and two subtypes of inhibitory interneuron (PV and SOM) populations. PLI measures phase synchronization (see Materials and Methods) [44], with values ranging 0–1. A PLI of 0.0 indicates the absence of coupling between the responses of a specific subtype of inhibitory interneurons and those of the Pyr population, whereas a PLI of 1.0 represents perfect phase locking with a constant, nonzero phase delay. A PLI closer to 1 indicates strong nonzero phase locking. We summarized PLI values between Pyr and two subtypes of inhibitory interneuron (PV and SOM) populations for frequencies ranging 10–80 Hz in steps of 5 Hz, as a function of E/I ratio caused by changes in Pyr and PV population sizes (S8A Fig). In the frequency range where the dPLI between Pyr and PV populations was 0.5, the PLI was approximately 0.0. When the E/I ratio was increased from 3.5:1.0, the PLIs between Pyr and PV populations tended to be enhanced at peak frequencies around 10–15 Hz, 25–30 Hz, and 40–45 Hz (see also Fig 3B). PLIs between Pyr and the two subtypes of inhibitory interneuron populations as a function of E/I ratio caused by altering Pyr and SOM population sizes are summarized in S8B Fig. When the E/I ratio exceeded 3.5:1, the PLI values between Pyr and PV populations tended to increase with frequency. Additionally, the frequencies of the peaks in the PSTH power around 10–15 Hz and 40–45 Hz (Fig 4B) appeared to correspond to strong PLI values, at least in part. These results suggest that interactions between Pyr and PV populations play an important role in determining the peaks of PSTH power in the frequency range.

## Discussion

In people with ASD and SZ, the dysfunction of multiple neural systems, including atypical visual perception, is hypothesized to stem from an E/I imbalance in the brain [18–28]. In the present study, to investigate the roles of specific subtypes of inhibitory interneurons in neural system dysfunction, we developed a microcircuit model of layers 2/3 in the visual cortex with a biologically plausible structure based on excitatory Pyr neurons and three subtypes of inhibitory interneurons (PV, SOM, and VIP), which represent a functional microcircuit of V1, such as orientation selectivity. By using this model, we found that when E/I ratio were increased by decreasing the PV population, the neuronal activity patterns of PV interneurons preceded those of Pyr neurons (Fig 5A), which enhanced beta (20–30 Hz), low-gamma (30–60 Hz), and high-gamma (> 60 Hz) range oscillations (Fig 3). By contrast, Pyr neuronal activity was the precursor to PV activity when E/I ratios were increased by decreasing the SOM population (Fig 5B), which preferentially impaired neuronal activity at gamma frequency (Fig 4). If changes in the E/I ratio resulting from shifts between specific subtypes of inhibitory and Pyr populations within the functional microcircuit are a common characteristic or structural feature of the V1 or cortical areas in patients with ASD, the modulation of information flow suggested by our model simulations may be widely observed in these areas.

### Possible mechanisms of cortical microcircuit activity modulation arising from E/I ratio disruption

**Fig 6:**
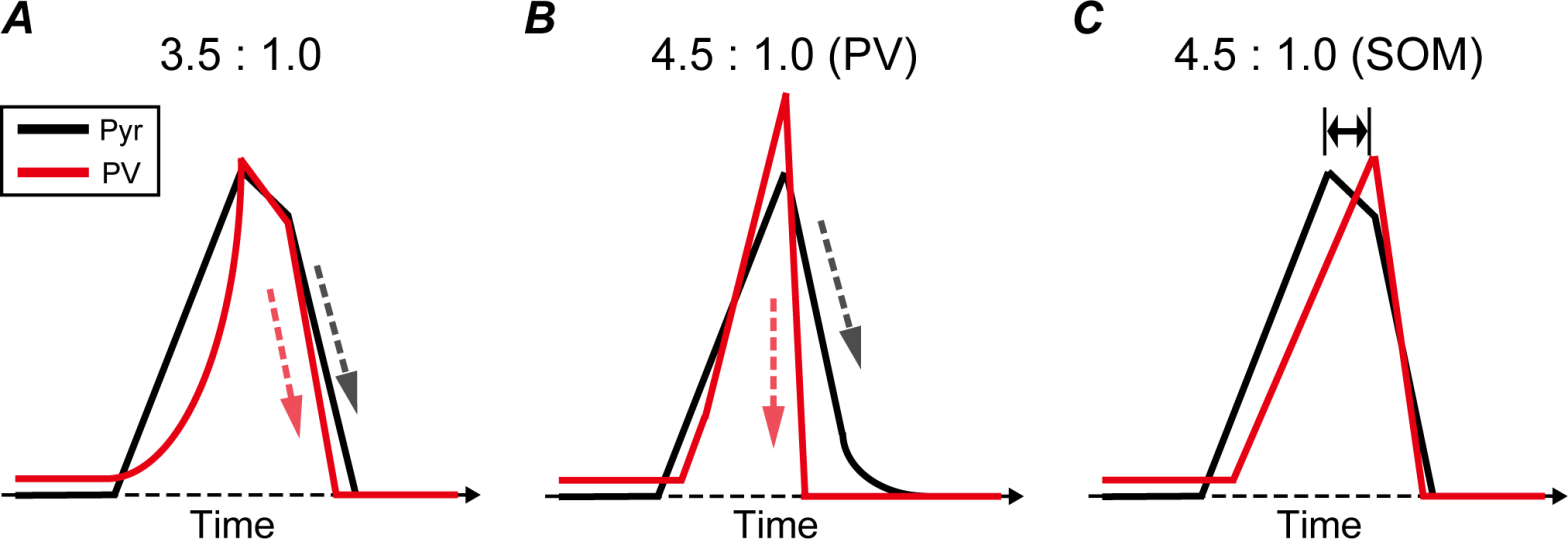
Modulations in activity dynamics in pyramidal (Pyr) neuron (black lines) and parvalbumin (PV) interneuron (red lines) populations by disrupting the excitatory/inhibitory (E/I) balance with an increased number of Pyr neurons. ***A.*** Typical pattern of the time course of neuronal activity in Pyr and PV populations with an E/I ratio of 3.5:1.0. Black and red dashed arrows represent the speed of decay for the Pyr and PV populations, respectively. Under this condition, activation in the Pyr neuron population preceded that of PV interneurons. However, the activity peaks of these populations were almost at the same time. The decay speed of the PV population seemed slightly faster than that of the Pyr population. ***B.*** Typical pattern of the time course of neuronal activity in Pyr and PV populations with an E/I ratio of 4.5:1.0 caused by a decrease in PV interneuron number. Conventions are the same as those in panel ***A*.** Under this condition, similar to the E/I ratio of 3.5:1.0 (panel ***A*)**, the peaks of these two populations seemed to be synchronized. However, the PV interneuron population activity decayed more rapidly than that of Pyr neurons (black and red dashed arrows, respectively), which might modulate neuronal firing at beta and gamma frequencies. ***C.*** Typical pattern of the time course of neuronal activity in Pyr and PV populations with an E/I ratio of 4.5:1.0 caused by a decrease in somatostatin (SOM) number. A decrease in the number of SOM interneurons seemed to induce a delayed activity peak in the PV population compared with the Pyr population (two-headed arrow), which might induce a distinct modulation of oscillatory activity in the microcircuit model compared with E/I ratio disruption arising from a change in PV interneuron numbers.

In our proposed network, spike responses from the excitatory Pyr population activated all subtypes of inhibitory interneurons (Fig 1). The typical pattern of dynamics, for PSTHs representing the temporal population activity of Pyr neurons and PV interneurons with an E/I ratio of 3.5:1.0, are illustrated in Fig 6A. Under this condition, the Pyr population activated the PV population and was then suppressed by the activated PV population. This interaction between Pyr and PV populations are considered to underlie the generation of synchronized oscillations at the gamma band frequency in our microcircuit model (as has been proposed as the pyramidal-interneuron-gamma model [45,46]). In addition, we observed frequent overlaps in the peaks of PSTHs for Pyr neurons and PV interneurons with a bin size of 2.0 ms.

The dPLI analysis demonstrated that the neuronal activities of PV interneurons preceded those of Pyr neurons (Fig 5A) when the E/I ratio was increased by decreasing the number of PV interneurons. The typical patterns of PSTHs in Pyr and PV populations with an E/I ratio of 4.5:1.0 (caused by a change in the number of Pyr neurons and PV interneurons) are shown in Fig 6B. Under this condition, the population activity of PV interneurons dropped more rapidly after the peak (red dashed arrow in Fig 6B) compared with under conditions of a 3.5:1.0 E/I ratio (red dashed arrow in Fig 6A). This rapid drop of PV activity suggests a possible important role of PV interneurons as the precursor to Pyr population activity and for enhancing neuronal activity at higher frequency ranges. In addition, following a significant decrease in the activity of PV interneurons, Pyr neurons may be further inhibited by SOM interneurons. This inhibition of Pyr neurons by regular-spiking interneurons, such as SOM, might underlie the subtle modulation of the peaks in the beta and low-gamma frequencies with increasing E/I ratios [7,8].

In contrast to the model’s responses to different E/I ratios caused by decreasing the PV population (Fig 5A), Pyr neuron activity functioned as the precursor to PV interneuron activity when the E/I ratio was increased by decreasing the SOM population (Fig 5B). The typical patterns of PSTHs in Pyr neurons and PV interneurons for an E/I ratio of 4.5:1.0 (caused by changing the Pyr neuron and SOM interneuron numbers) are illustrated in Fig 6C. Notably, increasing the E/I ratio by decreasing the SOM population tended to markedly delay the activation of the PV population, and to further delay the peak in activity of the PV population compared with that of Pyr neurons (double-headed arrow in Fig 6C). These delayed peaks of PV activity under E/I disruption with a decreasing SOM population might be reflected in smaller intensity levels of dPLI (<0.5) between Pyr and PV populations with low- and high-gamma-frequency ranges (Fig 5B). Furthermore, an increase in the E/I ratio due to a reduction in the number of SOM interneurons led to increased population activity of PV interneurons, whereas the population activities of Pyr neurons and SOM interneurons were either suppressed or remained unmodulated (Fig 4A). The activation of the PV interneuron population may play a critical role in markedly shifting the peak locations of beta and low-gamma oscillations toward higher frequencies [7,8].

In our microcircuit model, PV interneurons recurrently connect within their population (Fig 1A). A previous computational study demonstrated that self-inhibition through recurrent connections among inhibitory neurons plays a critical role in generating and modulating oscillatory activity in neuronal networks [47]. In our present study, the impacts of recurrent connections among PV interneurons within the microcircuit may be influenced by altering the population sizes of specific interneuron subtypes, potentially contributing to determining and modulating the information flow direction between Pyr and PV populations. In addition, as suggested by Zhang et al. [47], recurrent connections within the PV interneuron population may modulate oscillatory responses in our microcircuit model. These recurrent connections among PV model interneurons might play a crucial role in linking directional flow modulation with oscillatory responses.

Simulations of our proposed microcircuit model revealed that the activity of the Pyr population consistently precedes that of the SOM interneuron population, irrespective of the E/I ratio and the population size of subtype interneurons (Fig 5). The potential mechanism underlying the role of Pyr neuron activity as a precursor to SOM interneuron activity may arise from differences in the membrane time constants across neuronal classes and subtypes in the model. The membrane time constants among Pyr neurons, SOM interneurons, and VIP interneurons were comparable (see Materials and Methods). Additionally, synaptic currents originating from the Pyr neuron population served as the primary input for activating the SOM interneuron population. By contrast, the membrane time constant of PV interneurons was markedly faster than that of the other neuronal classes and subtypes [4,31]. Interactions between the population size of PV interneurons and their characteristic fast-spiking properties may play an important role in modulating the direction of information flow between Pyr and PV populations. In addition to differences in membrane time constants, the PV and SOM interneuron populations exhibited distinct characteristics, such as the synaptic-decay time to the Pyr neuron population and the presence or absence of recurrent connections within their respective populations. The recurrent connections within inhibitory interneuron populations, in particular, may play an important role in the modulation of the information flow [47]. Interactions between these factors and population size may determine the direction of information flow between neuronal populations.

### Predictions from simulations of the proposed microcircuit model

E/I imbalance, resulting from disruptions in the ratio of excitatory-to-inhibitory neurons, may be an important factor contributing to neural system dysfunction in individuals with ASD [18,21,24–26,28]. Here, our simulation results implied the distinct impacts of PV and SOM inhibitory interneurons on the atypical temporal coordination of neuronal activity in cortical circuits due to the E/I imbalance. Furthermore, our analyses of the model’s responses using the dPLI method indicate that a disruption to the E/I ratio caused by changes in the numbers of excitatory Pyr neurons and specific subtypes of inhibitory interneurons leads to a marked modulation in the direction of flows between Pyr and PV populations (Fig 5), suggesting the distinct influences of PV and SOM inhibitory interneurons on the atypical temporal coordination of neuronal activity in cortical circuits (Figs 3 and 4). However, this hypothesis is based only on the simulation results of our computational model. To date, there is no support or evidence from physiological studies concerning the specific neuronal-subtype-dependent disruption of E/I ratios. However, this hypothesis may be testable by subtype specific inactivation. Therefore, further physiological and computational studies are required to investigate the directions of flows in various levels of neuronal networks and to deepen our understanding of the mechanisms underlying their dysfunction in pathological abnormalities.

### Responses of our model compared with those of previous pathological studies

In addition to our microcircuit model, several computational studies have investigated neural behaviors in pathological abnormalities. Park et al. [48] proposed a spiking network model comprising multiple neuronal groups with different topological properties in a macroscopic network. Their model suggested that locally dense connectivity decreased the complexity of neuronal network activity; this is similar to what has been reported in children with ASD who were aged between 2 and 24 months [49]. In addition, our previous computational study [35] investigated the influence of periodic external inputs to a network model with various E/I ratios (between excitatory and inhibitory neurons) on the modulation of neuronal dynamics. These previous computational studies with spiking neuronal networks have provided insights into the mechanisms underlying dysfunction in pathological abnormalities. However, they included only a single class of inhibitory neuronal population, and thus they cannot account for different mechanisms induced by E/I balance changes due to different interneurons (Figs 3 and 4).

In our simulations, increasing the E/I ratio by decreasing the SOM population induced synchronized activity with peaks at higher frequency levels but suppressed the strength of these oscillations (Fig 4). Similarly, a physiological study using data from magnetoencephalography (MEG) revealed that gamma band activity amplitudes are reduced when individuals with ASD perceive complex, ambiguous figures [16]. These findings imply that an increased E/I ratio caused by decreased SOM interneuron numbers may, at least in part, contribute to impaired neuronal activity in gamma band oscillations during perceptual organization in individuals with ASD.

Interactions among V1 microcircuits within and across receptive fields may play a critical role in modulating neuronal activity [9,50,51] and organizing orientation selectivity involving visual perception [40,41,52]. When microcircuits interact through inhibitory horizontal connections, dynamic competition often leads to the selective activation of a specific microcircuit while significantly suppressing the activity of others. This phenomenon is likely attributable to the enhancement of excitatory responses within each microcircuit [53]. Our model simulations indicated that increasing the E/I ratio by reducing the PV interneuron population resulted in the activation of the excitatory Pyr model neuron population, as well as the PV and SOM interneuron populations (Fig 3). The activation of Pyr model neurons through an increased E/I ratio resulting from a reduced PV interneuron population may facilitate dynamic competition among mutually inhibiting microcircuits. This, in turn, could drive the interacting microcircuits into unstable activation states, thereby impairing the establishment of selectivity and perception. A disruption of the E/I balance due to a reduction in PV interneurons may underlie the reduced global motion perception in patients with ASD [11,12].

### Other possible inputs to the cortical microcircuit in V1

In the layered network structure, layer 4 serves as the primary input station, receiving feedforward visual inputs and transmitting its activity to layers 2/3. By contrast, our microcircuit model of the layers 2/3 in V1 directly received the feedforward inputs representing visual stimuli. We applied a much higher connection probability to the excitatory Pyr neuron population than to the inhibitory interneuron population, with characteristics consistent with those found in a previous computational study [31]. However, physiological studies have found that synaptic connections mediating visual stimuli are more densely projected to the inhibitory interneuron population in the V1 than they are in our current model [54,55]. In the layered structure of V1, feedforward inputs representing visual stimuli project mainly onto the granular and deep layers [55]. In addition, inhibitory interneurons and excitatory neurons in the granular layers project their signals to the neurons in layers 2/3 [29,55,56], potentially contributing to regulating neuronal activity in layers 2/3 of V1. Modeling the cortical microcircuit of the granular layers in V1 as input to the layers 2/3 may be necessary to provide a more accurate representation and elucidate the impact of E/I imbalance in the visual cortex.

In the present study, two types of external inputs are projected to the proposed microcircuit model: background and feedforward inputs. However, the feedback signals from higher visual areas, such as V2 and V4, are thought to underlie the neuronal modulation in V1 [57]. Recent computational studies suggest that local disinhibition by connections from VIP to SOM interneuron populations may be a plausible mechanism for the top-down attentional modulation of responses in the cortical microcircuit in layers 2/3 of the V1 [31,33]. In these models, top-down attention is applied to the VIP population, leading to a marked activation of not only VIP but also Pyr and PV populations, while the SOM interneuron population is inhibited as a result of VIP activation. Modulation patterns of our present model by providing feedback signals to VIP population may resemble those of these previous models. The disinhibitory connection from VIP to SOM populations appears to play a critical role in the neuronal modulation of visual cortices [33]. By contrast, our proposed microcircuit model does not incorporate feedback signals from higher visual cortices that mediate top-down attention. The absence of these signals may lead to significant suppression of VIP interneuron activity by the feedforward inputs, in contrast to the activation of Pyr neurons and other subtypes of interneurons (Fig 2).

V1 responses are significantly modulated not only by visual stimuli projected onto their receptive fields but also by the contextual structures surrounding them [58]. Horizontal connections across receptive fields have been proposed as the underlying mechanism of such surround modulation [50,51]. In the cat V1, many excitatory neurons in superficial layers have axons that project horizontally over large distances [59–61], which may contribute to this modulation of visual activity by surrounding stimuli [62]. However, our current model does not include interactions between microcircuits with different receptive fields. Additionally, interactions among microcircuits sharing receptive fields appear critical for establishing orientation selectivity and perception [40,41,52]. By contrast, the current model lacks such interactions within receptive fields. Extending the model to include additional inputs to layers 2/3 of V1, such as feedback signals, horizontal connections across receptive fields, and neural interactions within receptive fields, may provide further insights into the mechanisms underlying dysfunction in pathological conditions.

### Possible impacts of the E/I imbalance on the interactions among microcircuits

In the present study, to simplify the simulation conditions and analyze network responses and information flow between neuronal classes, we did not incorporate interactions among microcircuits, which represent a functional unit of V1. However, as discussed previously, such interactions among microcircuits within V1 may underlie the modulation of neural responses and visual perception [9,41]. These interactions appear to be mediated by dynamic competition between mutually inhibiting microcircuits [40,41]. During this competition, the activity of these mutually inhibited microcircuits is strongly influenced not only by the strength of the intermicrocircuit connections but also by the strength of the intramicrocircuit connections [53]. Notably, when intermicrocircuit inhibition dominates self-inhibition within a microcircuit, a specific microcircuit tends to persist in the interaction, exhibiting winner-take-all behavior. In the present study, we varied the E/I ratios by altering the population sizes of different subtypes of interneurons while maintaining the total neuron count constant. This approach may also influence the strength of self-inhibition within the microcircuit. E/I imbalance within V1 microcircuits possibly disrupts responses in V1 or other visual cortices by altering signal transmission and intermicrocircuit interactions.

Developing a model that incorporates interactions between microcircuits is essential for deepening our understanding of how E/I imbalance contributes to neural system dysfunction. However, the precise structures and specific interneuron subtypes involved in mediating microcircuit interactions within and across classical receptive fields in V1 remain poorly understood, which may be mediated by short- and long-range horizontal connections, respectively [63]. In addition, recent physiological study has reported various modulation patterns of excitatory neurons and three inhibitory interneuron subtypes depending on the presented stimuli [58]. The association between neuron-class-specific modulations and presented stimuli may provide important insights into the structure of the horizontal connection between microcircuits in V1. To elucidate the impact of E/I imbalance on neural system dysfunction, further studies, and knowledge are needed to investigate and model the structural organization of V1 microcircuit interactions within and across classical receptive fields during typical development.

### Various patterns of E/I imbalance in the brain

We computationally investigated the relationships between E/I imbalances and atypical neuronal network structures associated with neural system dysfunction. To simplify the representation of the E/I imbalance in the cortical microcircuit model, we varied the E/I ratios by altering the population size of different interneuron subtypes while keeping the total neuron count constant. However, previous studies suggest that the E/I imbalance arising from synaptic weight alterations might underlie dysfunction in SZ patients [19,23]. Notably, a physiological and computational study revealed that extra-large synapses between excitatory neurons, which induce significant excitatory post-synaptic potentials (EPSPs), were observed more frequently in the brains of SZ patients than in those of controls [23]. This characteristic distribution of excitatory synapses in SZ brains could be modeled by adjusting the parameters of the log-normal distribution used to describe the synaptic connectivity between Pyr model neurons (Eq. (8)). By contrast, a Gaussian distribution may not be appropriate for modeling synaptic distributions including extra-large synapses. An increase in extra-large synapses among the Pyr neurons may induce distinct modulation patterns compared with the E/I ratio disruptions caused by changes in the neuronal population size in the model. This disruption occurs because Pyr population activation can alter the direction of information flow within the microcircuit. Further studies to examine the interactions between neuronal population size and synaptic weight in relation to E/I ratio changes are needed to elucidate further the mechanisms underlying dysfunction under pathological conditions.

As previously discussed, the distribution of synaptic strength may play a critical role in determining the E/I balance in the brain. However, visual cortical plasticity may be reduced in patients with ASD [64]. E/I ratio disruption may occur independently of the synaptic strength regulation induced by synaptic plasticity.

In this study, synaptic-decay time constants were common to our earlier microcircuit diagram based on physiological and computational studies (Materials and Methods section) [2,29–31,33]. The synaptic-decay time constant may serve as one of the factors that determine the E/I balance in the brain, as well as the distribution of synaptic strengths. Our previous study demonstrated that beta-band activity was markedly enhanced as the synaptic strength from Pyr neurons to PV interneurons decreased [33]. In contrast, gamma-band activity increased with reduced synaptic strength from Pyr neurons to SOM interneurons. Similarly, shorter (or longer) synaptic-decay time constants from Pyr neurons to PV or SOM interneuron populations may induce modulation patterns resembling those elicited by weaker (or stronger) synaptic strengths.

We defined the E/I ratio as the population size of Pyr excitatory neurons to the combined population size of all inhibitory interneuron subtypes (Table 1) [35]. Under these conditions, modifying the E/I ratio between Pyr and PV populations by adjusting their relative sizes concomitantly alters the E/I ratio between Pyr and SOM populations. PV interneurons are recurrently connected, providing self-inhibition, whereas SOM interneurons project to all other neuronal populations. The influence of these interneuron-subtype-specific network structures on the microcircuit may depend on their relative population sizes and the interactions between the Pyr-to-PV and Pyr-to-SOM ratios. In addition, recent computational study has reported the contribution of interneuron subtype to the control of trial-by-trial output variability in the cortical microcircuit [65]. Especially, this study indicated smaller trial-by-trial output variability when more pronounced feedforward inputs were provided to the PV population than the SOM population. Concomitant changes in the Pyr-to-PV and Pyr-to-SOM ratios induced by E/I imbalance may also contribute to trial-by-trial variability in the output of the microcircuit model, potentially underlying visual perceptual abnormalities observed in patients with ASD. Analyzing the combined effects of variations in Pyr-to-PV and Pyr-to-SOM ratios could provide further insight into the relationship between E/I imbalance and atypical neural activity.

Here, we applied a long-tailed, log-normal distribution to the strengths of synaptic conductance within the Pyr population [38,39]. By contrast, other types of synaptic conductance (excitatory-to-inhibitory, inhibitory-to-excitatory, and inhibitory-to-inhibitory synapses) follow Gaussian distributions [33–36,66,67]. However, the synaptic distributions may depend on the classes and subtypes of both presynaptic and postsynaptic neuronal populations. Notably, a physiological study revealed that the strength of both excitatory inputs to PV interneurons and their inhibitory inputs to excitatory neurons follow a log-normal distribution [68]. These neuron class-specific distributions of synaptic strength may influence the E/I balance within the neuronal network. The distribution of synaptic weights used to construct the network plays a critical role in determining the response properties and dynamic behavior of the microcircuit model [34,35]. However, as previously discussed, the impact of the synaptic weight distribution on the results observed in the present model simulations may be limited, as neuron-class- and subtype-specific membrane time constants could play a key role in modulating the model’s dynamic behaviors under conditions of the E/I balance disruption.

### The relationship between dPLI and PLI analyses and oscillatory neuronal responses with respect to the direction of information flow

In this study, we used the dPLI and PLI to analyze the direction of information flow between Pyr and PV or SOM populations. This metric characterizes the pattern of phase synchronization associated with the direction of information flow, based on the asymmetry in the distribution of phase differences within a given time window [44]. If significant phase synchronization exists and a consistent phase lag (i.e., a delay in synchronization) is maintained between two neuronal populations, this metric is effective for evaluating information flow within the network. The specific characteristics of the dPLI and PLI depending on phase synchronization appear to have contributed effectively to the analysis of response dynamics in our microcircuit model. This is particularly relevant given that the model exhibited more marked oscillatory activity in response to feedforward inputs compared to that observed in biological experiments. Nevertheless, even in the absence of strongly oscillatory responses as observed in our study, the dPLI and PLI methods may still be applicable to weakly oscillatory activity that retains some degree of synchronization, even under more asynchronous conditions. Importantly, under such conditions, asynchronous components may be attenuated through temporal averaging within a given time window, which could reduce the value of these metrics but may not reverse the direction of information flow. Additionally, oscillatory activity has been observed in the visual cortex [69,70], although it appears less prominent than that observed in our model. The proposed microcircuit model may be utilized as an abstract framework for simulating such oscillatory neuronal dynamics.

### Limitations and possible development of our microcircuit model

Simulations of our proposed microcircuit model revealed significant oscillatory responses when feedforward inputs modeled as Poisson spike trains at 25 Hz were applied (Fig 2). In biological studies, including those of electrophysiology and psychophysics, environmental noise may influence neuronal activity. However, to simplify the simulation conditions, our microcircuit model received only background inputs that induced spontaneous activity and feedforward inputs that represented visual stimuli without incorporating other inputs, such as environmental noise. Furthermore, as previously discussed, additional inputs to layers 2/3 of V1, such as feedback signals, horizontal connections across receptive fields, and neural interactions within receptive fields, may help reproduce biologically plausible responses. Examining model responses with these additional inputs could provide further insight into the mechanisms underlying neuronal oscillations.

In the present study, we used the PSTH of Pyr neurons as a metric of neuronal signals, similar to previous computational studies [31,71]. By contrast, physiological responses of ASD and SZ patients have been recorded using MEG [16,17]. In addition, a previous study proposed an algorithm for quantifying functional E/I ratios from neuronal oscillations [22], which was then applied to electroencephalogram data from typically developing subjects and children with ASD. The PSTH based on Pyr neuron activity from the present study might partially capture the characteristics of these non-invasive brain measurement signals. However, further analyses of a detailed model of MEG and electroencephalogram signals will be necessary to further understand the association between the E/I imbalance and atypical neural activities.

Previous studies have reported visual perception abnormalities in patients with ASD and SZ [72–75]. In addition, psychophysical analyses of the atypical characteristics of visual attention in patients with ASD may provide insights into the neural mechanisms [76,77]. However, we did not evaluate how the modulation of activity in our microcircuit model (by disrupting the E/I ratio) was reflected in dysfunctional visual perception or atypical characteristics of attention. Further studies comparing the characteristics of perception observed in psychophysical experiments with the activity of our cortical microcircuit model and its disrupted E/I ratios are therefore necessary to better understand the mechanisms of dysfunction in pathological abnormalities. Additionally, comparing model activities to visual perception requires incorporating interactions between microcircuits sharing receptive fields and feedback signals from higher visual cortices [41].

## Conclusions

To investigate the association between the E/I imbalance and atypical neural activities, we performed simulations of a biologically plausible network model with different E/I imbalances, which were induced by changing different numbers of PV or SOM interneurons to become Pyr neurons (or vice versa). Simulations with our model indicated that inducing an E/I balance disruption by decreasing PV interneuron numbers led to enhanced neuronal firing at beta and gamma frequencies in the cortical microcircuit. By contrast, disrupting the E/I balance by decreasing the SOM population preferentially impaired gamma-frequency activity. Furthermore, deeper analyses of the model’s responses suggested that E/I ratio disruptions that are induced by changing the numbers of specific interneuron subtypes can markedly modulate the direction of neural transmissions between Pyr and PV populations. Our results may contribute to understanding the distinct influences of PV and SOM inhibitory interneurons on the atypical temporal coordination of neuronal activity in functional cortical circuits. Our cortical microcircuit model offers important insights into the atypical structure of neuronal networks and the neural system dysfunction arising from E/I imbalances.

## Materials and methods

*Architecture of the control microcircuit model with an E/I ratio of 3.5:1.0*

In the present study, to investigate the influence of changes in E/I ratios due to specific neuron subtypes on neuronal modulation, we developed a computational microcircuit model with biologically plausible visual cortical layers 2/3 that combined excitatory Pyr neurons and three inhibitory interneuron subtypes (PV, SOM, and VIP) (Fig 1) by extending our previous model [33,34]. The architecture of our microcircuit is illustrated in Fig 1A. This model corresponds to a functional unit in the V1, such as that of orientation selectivity. To avoid increasing the complexity of analyzing network responses and to investigate the association between E/I imbalance and atypical neural activities, we followed a previous computational study and excluded interactions among microcircuits within or across their classical receptive fields in the current model [34]. The connectivity was adapted from earlier circuit diagrams based on physiological and computational studies [2,29–31,35].

The structure and connection probabilities of the current model were the same as those of our previous microcircuit model [33,34]. However, we increased the numbers of model neurons from the previous model to preserve biologically plausible activity under the different E/I ratios [35]. The full network of our model for the control responses consisted of around 13,000 integrate-and-fire neurons, divided into 10,341 excitatory Pyr neurons and 2,916 inhibitory interneurons, in accordance with previous computational studies [29,40,41] (Table 1). The E/I ratio of the model used to measure control responses was approximately 3.5:1.0. There were 1,341 PV, 875 SOM, and 700 VIP inhibitory interneurons in our control model. We determined the relative numbers for each neuronal class and subtype based on data reported from physiological experiments [2,78] and a previous computational model [30]. Detailed methods with respect to the population levels of these neuronal classes and subtypes are provided in Lee et al. [30] and Wagatsuma et al. [33,34].

The network parameter details are listed in Table 2 [2,4,55,56,79]. The connection probabilities were consistent throughout all simulations in this study, irrespective of E/I ratios. We determined the connectivity from the excitatory Pyr population to the three interneuron subtype populations based on the structure of layers 2/3 in a previous network model [30]. The connections from the three inhibitory interneuron subtypes to Pyr neurons were determined using the connection probabilities reported in Pfeffer et al. [2] as weighting factors (Table 2). We refer the reader to our previous study [33,34] for the detailed neuronal connectivity methods.

**Table 2:** Parameters of the microcircuit model. The structure of this model was based on our previous model [33,34].

Connection Probabilities	
**Excitatory-to-Excitatory**	0.1009
**Excitatory-to-Inhibitory**	0.1346
**Inhibitory-to-Excitatory**	0.1689
**Inhibitory-to-Inhibitory**	0.1371
**Weighting factors for neuron-class-specific connections**	
**Inhibitory-to-Excitatory**	PV-Pyr: SOM-Pyr = 1: 1
**Inhibitory-to-Inhibitory**	PV-PV: SOM-PV: VIP-SOM: SOM-VIP = 1: 0.857: 0.625: 1

We determined the connection probabilities and weighting factors according to Potjans et al. [29] and Lee et al. [30], respectively. These parameters are based on experimental data from a range of animal species [2,4,55,56,79]. We refer the reader to our previous study [33,34] for detailed methods with respect to neuronal connectivity. Abbreviations: PV, parvalbumin; Pyr, pyramidal; SOM, somatostatin; VIP, vasoactive intestinal polypeptide.

In our model, we applied a long-tailed, log-normal distribution to the distribution of the strengths of synaptic conductance within the Pyr population [35–39]. The strengths of other types of synaptic conductance (excitatory-to-inhibitory, inhibitory-to-excitatory, and inhibitory-to-inhibitory synapses) were distributed according to Gaussian distributions. The details of synaptic parameters are described in the following subsections.

### Cortical microcircuit models for a variety of E/I ratios

We performed simulations of the microcircuit model with different E/I ratios caused by converting specific subtypes of inhibitory interneurons to excitatory Pyr neurons, and vice versa. The numbers of Pyr neurons and each interneuron subtype for the modified microcircuits are listed in Table 1. Note that the total number of model neurons and the connection probabilities remained consistent throughout all simulations, irrespective of E/I ratio. The number of synapses depended on the numbers of presynaptic and postsynaptic neuronal populations.

### Model neurons and synapses

We developed the microcircuit model using integrate-and-fire neurons to represent all classes and subtypes of model neurons [32–34,80,81]. The dynamics of the subthreshold membrane potential (*V*) of a model neuron were calculated using the following equation:


$$\frac{dV(t)}{dt}\;=\;-\frac{V(t)-E_{l}}{\tau_{m}}\;+\;\frac{I_{Pyr}(t)+I_{PV}(t)+I_{SOM}(t)+I_{VIP}(t)+I_{ext}(t)}{C_{m}}$$ (1)

where *τ*_*m*_ is the membrane time constant and *C*_*m*_ is the membrane capacitance. *E*_*l*_ represents the leak-reversal potential. The neuronal model parameters are summarized in Table 3, and were chosen based on previous studies [4,31,80]. We used *V*_*thr*_ = −50 mV as the spike threshold. After spiking, we reset the membrane potential *V* to *V*_*reset*_ = −60 mV. *I*_*Pyr*_*(t), I*_*PV*_*(t), I*_*SOM*_*(t)*, and *I*_*VIP*_*(t)* represent the synaptic currents flowing into the model neurons from Pyr neuron class, PV, SOM, and VIP interneuron subtypes, respectively. *I*_*ext*_*(t)* refers to external inputs including background and feedforward inputs.

**Table 3:** Parameters of model neurons representing specific classes and subtypes.

		Parameter			
		Pyr	PV	SOM	VIP
***τ***_***m***_	Membrane time constant (ms)	10.5	3.1	11.8	10.9
***τ***_***ref***_	Refractory period (ms)	2.0	1.0	1.0	1.0
***C***_***m***_	Membrane capacitance (pF)	200			
***E***_***l***_	Leak-reversal potential (mV)	-70			

Abbreviations: PV, parvalbumin; Pyr, pyramidal; SOM, somatostatin; VIP, vasoactive intestinal polypeptide.

Synaptic currents from excitatory Pyr neurons, *I*_*Pyr*_*(t)*, were mediated by α-amino-3-hydroxy-5-methyl-4-isoxazolepropionic acid (AMPA)-type currents [33,34,80,82], and were calculated as:


$$I_{\;Pyr}(t)\;=\;g_{j}^{Pyr}\left(V(t)-V_{E}\right)\sum_{j}s_{j}^{Pyr}(t)$$ (2)

where *V*_*E*_ = 0 mV represents the reversal potential for excitatory synapses, and where *V* is the subthreshold membrane potential of a model neuron (see also Eq. 1). The conductance of the fully activated synapse *g*^*Pyr*^ is the receptor-specific conductance. The strength of *g*^*Pyr*^ within the Pyr population followed a log-normal distribution, whereas the strength of synaptic conductance from Pyr neurons to inhibitory interneurons followed Gaussian distributions [35–37]. Physiological studies have shown that the distribution of excitatory postsynaptic potential amplitudes between cortical Pyr neurons is well fitted to a log-normal distribution [38,39]. This log-normal distribution of synaptic strength among excitatory neurons plays a critical role in shaping the spatiotemporal characteristics of neural activity and the functional organization of cortical networks [66,67]. The fraction of open channels of model neurons from the *j*-th Pyr neuron, $s_{j}^{Pyr}$, was calculated as:


$$\frac{ds_{j}^{Pyr}}{dt}\;=\;-\frac{s_{j}^{Pyr}(t)}{\tau_{Pyr}}+\sum_{k}\delta \left(t-t_{j}^{k}-d_{j}\right)$$ (3)

where $\tau_{Pyr}$ represents the postsynaptic-decay time constant. We used $\tau_{Pyr}$ = 2.0 ms irrespective of the postsynaptic neuron class and subtype. The sum over *k* ran over all spikes from the connecting Pyr neurons. Each spike was entered as a Dirac delta function, δ(t), assuming a nonzero value at the spike times of the driven input neurons ($t_{j}^{k}$) (zero elsewhere) and integrating to unity over any interval that included $t_{j}^{k}$. $d_{j}$ is the delay from the *j*-th Pyr neuron, which obeyed a Gaussian distribution with a mean of 2.0 ms and a variance of 0.2 ms.

The membrane potentials of postsynaptic model neurons were reduced by receiving synaptic currents from the three subtypes of inhibitory interneurons. Synaptic currents from inhibitory model interneurons *I*_*Inh*_ were calculated as:


$$I_{\;Inh}(t)\;=\;g_{j}^{Inh}\left(V(t)-V_{I}\right)\sum_{j}s_{j}^{Inh}(t)$$ (4)

where the superscript or subscript *Inh* means the subtype of inhibitory neuron (PV, SOM, or VIP). The reversal potential for inhibitory synapses in *V*_*I*_ was set to −70 mV. The synaptic conductance of a fully open synapse of each specific subtype of inhibitory interneuron, *g*^*Inh*^, depended on the connections between neuron classes (Table 4) [31,83]. The distributions of synaptic conductance, *g*^*Inh*^, for inhibitory synapses followed a Gaussian distribution in accordance with previous computational studies [35–37,66,67]. The fraction of open channels in a PV, SOM, or VIP synapse, $s_{j}^{Inh}$, was calculated as:

**Table 4:** Postsynaptic parameters for synaptic conductance and decay time constants. These parameters were determined by classes and subtypes between presynaptic and postsynaptic neurons.

	From			
**Synaptic conductance *g* (nS), mean ± SD**				
**To**	**Pyr**	**PV**	**SOM**	**VIP**
**Pyr**	See main text	4.32 ± 0.432	1.26 ± 0.126	–
**PV**	0.95 ± 0.095	3.51 ± 0.351	1.22 ± 0.122	–
**SOM**	0.29 ± 0.029	–	–	0.32 ± 0.032
**VIP**	0.27 ± 0.027	–	1.18 ± 0.118	–
**Synaptic-decay time constants *τ***_***d***_ ** (ms)**				
**Pyr**	2.0	6.4	13.1	–
**PV**	2.0	4.6	5.2	–
**SOM**	2.0	–	–	13.1
**VIP**	2.0	–	10.2	–

Abbreviations: PV, parvalbumin; Pyr, pyramidal; SD, standard deviation; SOM, somatostatin; VIP, vasoactive intestinal polypeptide.


$$\frac{ds_{j}^{Inh}}{dt}\;=\;-\frac{s_{j}^{Inh}(t)}{\tau_{Inh}}+\sum_{k}\delta \left(t-t_{j}^{k}-d_{j}\right)$$ (5)

where $\tau_{Inh}$ represents the postsynaptic-decay time constant. These decay time constants were also determined in accordance with the connections between neuron classes or subtypes (Table 4) [2,31]. As shown for Pyr neurons in Eq. 3, the sum over *k* was over spike time ($t_{j}^{k}$); here, these were the times at which spikes occurred in the inhibitory interneurons.

The synaptic parameters for constructing our microcircuit model are summarized in Table 4, and are based on estimates of neuron-class-specific or neuron-subtype-specific postsynaptic currents [2,30,31,83]. However, to reduce neuronal activity to within a physiologically realistic range, we reduced the magnitudes of synaptic conductance *g* in the current model from those of our previous study [33,34] because of the larger scale of the model network [29].

In our network model, we applied external inputs *I*_*ext*_*(t)* of background inputs (for inducing spontaneous activity) and feedforward inputs (originating with visual stimuli) to all model neuron populations. The synaptic currents *I*_*ext*_*(t)* in Eq. 1 were given by AMPA-type synapses. The parameters and connection probabilities for these external inputs are summarized in Table 5.

**Table 5:** Parameters of background and feedforward inputs to the microcircuit model.

		Parameter			
		Pyr	PV	SOM	VIP
**Background inputs to each neuron class mediated by AMPA synaptic currents**					
***g***	Conductance (nS)	10.0			
***τ***_***d***_	Decay time constant (ms)	2.0			
***ν***_***BG***_	Rates (Hz)	190	770	100	150
**Feedforward inputs to each class of neuron population mediated by AMPA synaptic currents**					
# of fibers		500			
Connection probability		0.1	0.01	0.01	0.01
***g***	Conductance (nS)	2.5			
***τ***_***d***_	Decay time constant (ms)	2.0			
***ν***_***FF***_	Rates (Hz)	25			

Abbreviations: AMPA, α-amino-3-hydroxy-5-methyl-4-isoxazolepropionic acid; PV, parvalbumin; Pyr, pyramidal; SOM, somatostatin; VIP, vasoactive intestinal polypeptide.

To induce spontaneous activity, we applied cell-class-specific background inputs to all model neurons, which were described by an independent Poisson spike train. In addition, to investigate the influence of E/I ratio disruptions on the temporal coordination of neuronal activity, we also provided feedforward inputs (described by an independent Poisson spike train) to the target model neuron populations. We set the population size for these feedforward inputs to 500 fibers, which were randomly connected to target model neurons according to the connection probabilities (see Table 5). Excitatory neurons in layer 4 of V1 may preferentially form denser synaptic connections with PV interneurons in layers 2/3, as reported by Campagnola et al. [54]. However, in the current model, to activate the excitatory Pyr neuron population within a physiologically plausible range, we applied a much higher connection probability for feedforward inputs to the Pyr population than to inhibitory interneuron populations, similar to the approach used in a previous computational study [31]. Additionally, to simplify the model structure, we excluded inhibitory feedforward inputs representing the projections from the inhibitory neuron population in layer 4 [29,30]. These background and feedforward inputs were implemented by AMPA-type glutamatergic receptors [33,34,80,82], and were calculated as:


$$I_{\;Input}(t)\;=\;g_{j}^{Input}\left(V(t)-V_{E}\right)\sum_{j}s_{j}^{Input}(t)$$ (6)

where *g*^*Input*^ means the conductance of the fully activated synapse for background or feedforward inputs. The fraction of open channels of model neurons from the *j*-th Poisson spike train, $s_{j}^{Input}$, was calculated as:


$$\frac{ds_{j}^{Input}}{dt}\;=\;-\frac{s_{j}^{Input}(t)}{\tau_{Input}}+\sum_{k}\delta \left(t-t_{j}^{k}-d_{j}\right)$$ (7)

where the postsynaptic-decay time constant for background and feedforward inputs was $\tau_{Input}$ = 2.0 ms, irrespective of the target neuron class. See also Eqs. (3) and (5) for the detailed descriptions of these equations.

### Distributions of strengths and postsynaptic currents delays

In our cortical microcircuit, synaptic parameters such as conductance *g* and decay time constant *τ*_*decay*_ were determined according to previous physiological and computational studies that estimated synaptic characteristics depending on neuronal subtypes and cell-subtype-specific connections [2,30,31] (Table 4). In our model, the distributions of synaptic conductance *g* for excitatory-to-inhibitory, inhibitory-to-excitatory, and inhibitory-to-inhibitory neuronal connections obeyed a Gaussian distribution, whereas the strengths of conductance between Pyr neurons were determined using a log-normal distribution [35–39], as follows:


$$p(x)=\frac{\exp\left[\frac{-{\left(\log x-\mu \right)}^{2}}{2\sigma^{2}}\right]}{\sqrt{2\pi }\sigma x}$$ (8)

where *x* represents the excitatory postsynaptic potential amplitudes as computed from the resting potential. The mean and variance are given by *σ* and *μ*, respectively. To induce spontaneous activity under the application of background inputs, we used *σ *= 1.0 and $\sigma^{2}=\mu -log(0.10)$, respectively. In addition, synapses between Pyr neurons were described using a standard model with decay time constant *τ*_*Pyr*_ = 2.0 ms (see also Eqs. (2) and (3)).

Previous computational studies have reported that neuronal activity and dynamics are modulated by synaptic delays [33,36]. In the present study, we applied a Gaussian distribution with mean *d*_*0*_ and variance *d*_*0*_/10 to the synaptic delays. *d*_*0*_ was set to 2.0 ms with respect to connections from excitatory Pyr neurons, and *d*_*0*_ was 1.0 ms from the three subtypes of inhibitory interneurons. Note that, to simplify the model, the mean and variance of synaptic delays were consistent, irrespective of inhibitory interneuron subtype.

### Numerical experiments

Numeric simulations of the proposed model were conducted using different E/I ratios caused by changing a specific subtype of interneuron to become Pyr neurons, and vice versa. We applied background inputs to all model neurons during the simulations to induce spontaneous activity. Furthermore, feedforward inputs mediating visual stimuli were projected to the neuronal network from 100 biological ms after the start of the simulations. Note that the model network simultaneously received these two types of external inputs when visual stimuli were activated.

We integrated the differential equations using a fourth-order Runge–Kutta algorithm with a time step of 0.1 ms. To ensure the reproducibility of model responses, we repeated 50 trials of each model simulation with a length of 3 biological s. To reduce the effect of transients, the first 500 ms of simulation data were always discarded. The code for the simulations was written in C programming language.

### Data analysis of microcircuit model responses

To investigate the influence of E/I ratio disruptions arising from changes in the numbers of Pyr neurons and specific subtypes of inhibitory interneurons on neuronal modulation, we computed the population rates of all classes and subtypes of the model neuron population as the representative activity of the network model. The mean population rates were computed by counting the total number of spikes in each neuron subtype in a range from 500 ms to 3 biological s, averaged over 50 trials.

To investigate the contribution of E/I ratio disruptions arising from a change in the number of a specific subtype of inhibitory interneurons to the modulation of the characteristics of synchronized activity in the V1, we used PSTHs of the Pyr neuron population in a range of simulation data from 500 ms to 3 s [33]. These PSTHs represent an estimate of the time-varying firing rates of the Pyr neuron population. They were computed by dividing the firing activity of the Pyr neuron population into discrete time bins of 2 ms and counting the number of spikes within each time bin. We analyzed the frequency characteristics of these PSTHs of the Pyr population with different E/I ratios. However, the total number of model neurons in our microcircuit model was consistent, irrespective of E/I ratio. The PSTHs of the Pyr neuron population were decomposed into frequency components using a fast Fourier transform, and the mean oscillation amplitude was calculated by averaging the frequency power amplitudes across all trials. Prior to the fast Fourier transform analysis, PSTHs of the Pyr population were smoothed using a Gaussian filter with a variance of 5 ms to minimize the influence of the periodic responses.

The PLI has been used as a pairwise measure of functional connectivity among cortical areas [84]; the dPLI indicates which of the two signals is leading and which is lagging in phase [44]. To assess the directional modulation of neuronal flows induced by disruptions in E/I ratios, we analyzed the dPLI from PV to Pyr and from SOM to Pyr populations for specific frequency bands. dPLI between two populations were computed in accordance with Stam and van Strassten [44].

To analyze the directional modulation of neuronal flows, the dPLI was computed using PSTHs of the Pyr neuron and PV or SOM interneuron populations with discrete time bins of 2 ms in a range of simulation data from 500 ms to 3 s. These PSTHs were standardized using Z-score and were filtered into the specific frequency band using a band-pass filter. We applied the Hilbert transform to these band-pass filtered signals for analyzing the instantaneous phase of these two signals involved. The analytical signal ψ(t) is considered to be complex with *s(t)* of a real time series and $\hat{s}(t)$ of the Hilbert transform of this real signal:


$$\psi (t)=s(t)+i\hat{s}(t)=A(t)e^{i\Phi (t)}$$ (9)

where Φ(t) is the phase. Hilbert transform of *s(t)* was calculated using the following equation:


$$\hat{s}(t)=\frac{1}{\pi }P.V.\int_{-\infty }^{\infty }\frac{s(t)}{t-\tau }d\tau$$ (10)

where *P.V.* represents the Cauchy principal value. The instantaneous phase Φ(t) at time *t* is given as follows:


$$\Phi (t)=arctan\frac{\hat{s}(t)}{s(t)}$$ (11)

From the instantaneous phase of two signals, the phase difference or relative phase ΔΦ(t) is computed as follows:


$$\Delta \Phi (t)=\Phi_{Inh}(t)-\Phi_{Pyr}(t)$$ (12)

where $\Phi_{Pyr}(t)$ and $\Phi_{Inh}(t)$ indicate instantaneous phase of Pyr and specific interneuron subtype populations, respectively. The dPLI from the specific interneuron subtype *Inh* to *Pyr* populations is defined as follows:


$${dPLI}_{Pyr}^{Inh}=\frac{1}{N}\sum_{t=1}^{N}H\left(\Delta \Phi (t)\right)$$ (13)

where *H* represents the Heaviside step function.

The dPLI is represented by an intensity value ranging between zero and one. In the present study, if the activity of the specific inhibitory subtype population for the specific frequency band preceded that of the Pyr population, the dPLI value was > 0.5. By contrast, a dPLI value < 0.5 indicated that Pyr population activity was the precursor of the specific inhibitory interneuron subtype population activity. Data analyses and visualizations were performed using Python programming language. A *t* tes*t* was conducted to determine whether the dPLI was significantly different from 0.5.

The PLI is a measure of phase synchronization. The PLI between the specific interneuron subtypes *Inh* and *Pyr* is defined as follows:


$${PLI}_{Pyr}^{Inh}=\left|\left\langle sign\left(\Delta \Phi (t)\right)\right\rangle \right|$$ (14)

where *sign* represents the sign function, and | | and <> denote the absolute and average values, respectively. The relationship between PLI and dPLI can be expressed as follows:


$${PLI}_{Pyr}^{Inh}=2.0\;\times \;\left|0.5-\;{dPLI}_{Pyr}^{Inh}\right|.$$ (15)

The PLI ranged from 0 to 1. A PLI of 0.0 indicates the absence of coupling between the responses of a specific subtype of inhibitory interneurons and those of the Pyr population. Conversely, a PLI of 1.0 represents perfect phase locking at a constant, nonzero phase delay. A PLI closer to 1 indicates strong nonzero phase locking.

## Supporting information

S1 Fig Raster plots in the proposed microcircuit model with a ratio of excitatory to inhibitory neurons of 3.0:1.0 with respect to the sizes of Pyr and PV populations. As shown in Fig 2A, raster plots showing all spike trains of the pyramidal (Pyr), parvalbumin (PV), somatostatin (SOM), and vasoactive intestinal polypeptide (VIP) neuron populations for 1,500 ms. Spikes of excitatory Pyr neurons and the three subtypes of inhibitory interneurons are illustrated by black and gray dots, respectively. For this plot, visual stimuli were given from 0 ms.
(TIF)

S2 Fig Raster plots in the proposed microcircuit model with a ratio of excitatory to inhibitory neurons of 4.0:1.0 with respect to the sizes of Pyr and PV populations. Conventions are same as those in Fig 2A in the main text and S1 Fig.
(TIF)

S3 Fig Raster plots in the proposed microcircuit model with a ratio of excitatory to inhibitory neurons of 4.5:1.0 with respect to the sizes of Pyr and PV populations. Conventions are same as those in Fig 2A in the main text and S1 Fig.
(TIF)

S4 Fig Raster plots in the proposed microcircuit model with a ratio of excitatory to inhibitory neurons of 3.0:1.0 with respect to the sizes of Pyr and SOM populations. As shown in Fig 2A in the main text, raster plots showing all spike trains of the Pyr, PV, SOM, and VIP neuron populations for 1,500 ms. Conventions are same as those in Fig 2A in the main text.
(TIF)

S5 Fig Raster plots in the proposed microcircuit model with a ratio of excitatory to inhibitory neurons of 4.0:1.0 with respect to the sizes of Pyr and SOM populations. Conventions are same as those in Fig 2A in the main text.
(TIF)

S6 Fig Raster plots in the proposed microcircuit model with a ratio of excitatory to inhibitory neurons of 4.5:1.0 with respect to the sizes of Pyr and SOM populations. Conventions are same as those in Fig 2A in the main text.
(TIF)

S7 Fig Analyses of directed phase lag index (dPLI [44]) for excitatory/inhibitory (E/I) ratios with respect to pyramidal (Pyr) and parvalbumin (PV) populations (A) and Pyr and somatostatin (SOM) populations (B) for frequency bands from 10 to 80 Hz (in steps of 10 Hz). Conventions are same as those in Fig 5 in the main text. **A.** Values of dPLI from PV or SOM inhibitory interneurons to Pyr neurons as a function of E/I ratio with respect to Pyr and PV populations. ***B.*** Values of dPLI from PV and SOM to Pyr populations as a function of E/I ratios with respect to Pyr and SOM populations. The modulation patterns of dPLI values associated with the PSTH power peaks in the 10–20 Hz, 20–30 Hz, and 40–50 Hz frequency bands (Figs 3 and 4 in the main text) resemble those observed in other frequency ranges.
(TIF)

S8 Fig Analyses of the phase lag index (PLI [44]) for excitatory/inhibitory (E/I) ratios for pyramidal (Pyr) and parvalbumin (PV) populations (A) and Pyr and somatostatin (SOM) populations (B) for frequency bands 10–80 Hz (in steps of 5 Hz). Black circles and gray pentagons represent the mean PLI values from PV to Pyr and SOM to Pyr populations, respectively. PLI values from PV to Pyr and from SOM to Pyr populations for each trial are shown by small black and gray circles, respectively. ***A.*** PLI values from PV or SOM inhibitory interneurons to Pyr neurons as a function of the E/I ratio for Pyr and PV populations. The PLI values between the SOM and Pyr populations (gray pentagons) were almost constant, irrespective of the frequency band and E/I ratio. By contrast, when the E/I ratio was increased from 3.5:1.0, the PLI values between the Pyr and PV populations (black circles) tended to exceed 0.25 at peak frequencies around 10–15 Hz, 25–30 Hz, and 40–45 Hz (see also Fig 3B). ***B.*** PLI values from PV and SOM to Pyr populations as a function of the E/I ratio for Pyr and SOM populations. As shown by the E/I ratio for the PV population (panel A), the PLIs from the SOM to Pyr populations (gray pentagons) did not fluctuate around values of one. Additionally, when the E/I ratio exceeded 3.5:1, the PLI values between the Pyr and PV populations (black circles) tended to increase as the frequency increased. Furthermore, the frequencies of the peaks in the PSTH power around 10–15 Hz and 40–45 Hz (Fig 4B) appeared to correspond to strong PLI values, at least in part.
(TIF)
