# Supplementary figures and images for: Excitatory/inhibitory ratio disruption modulates neural synchrony and flow directions in a cortical microcircuit
Source: PLoS Comput Biol. 2025 Aug 6;21(8):e1013306. doi: 10.1371/journal.pcbi.1013306 (PMC12349708; doi:10.1371/journal.pcbi.1013306)

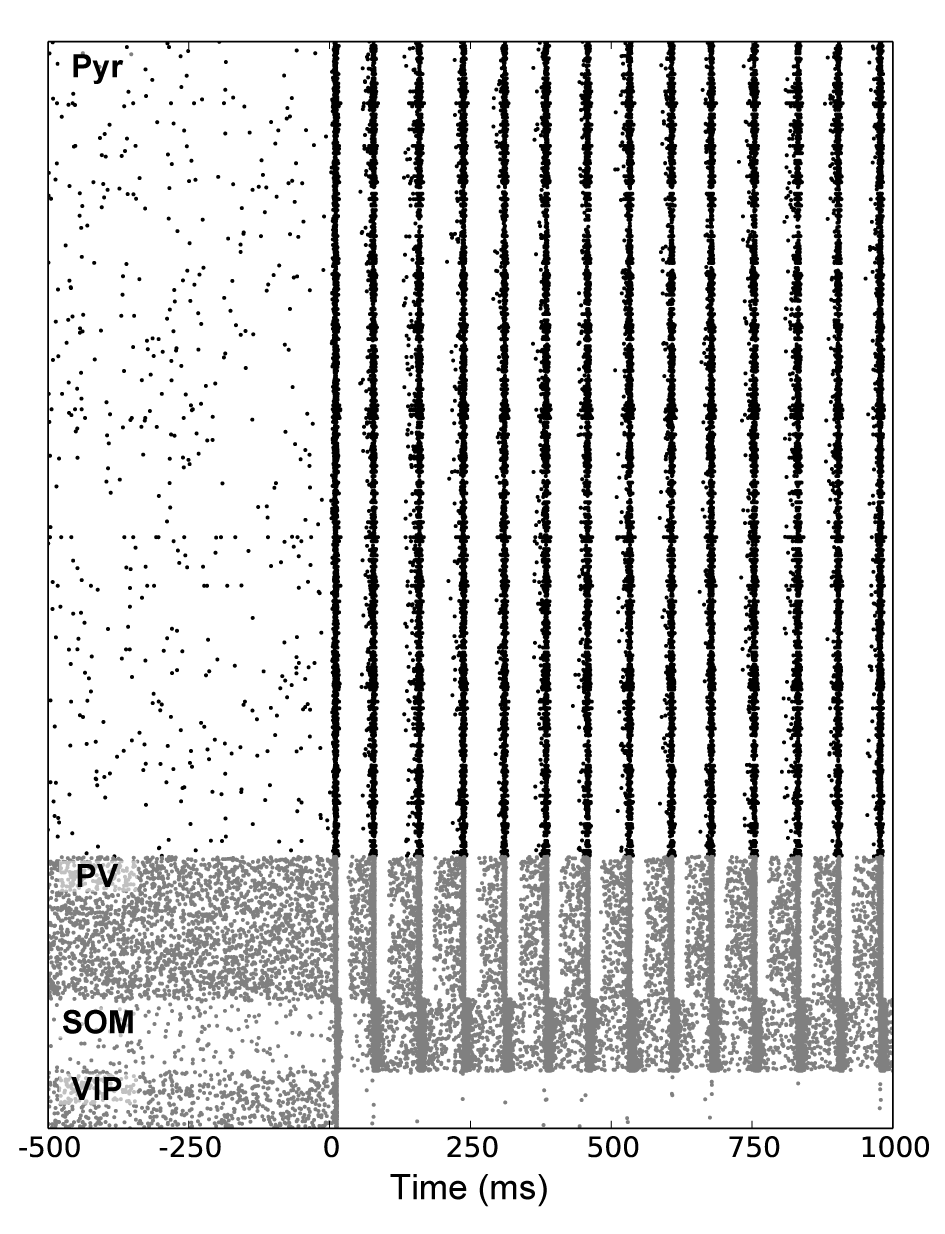

Supplement: S1 Fig — As shown in Fig 2A, raster plots showing all spike trains of the pyramidal (Pyr), parvalbumin (PV), somatostatin (SOM), and vasoactive intestinal polypeptide (VIP) neuron populations for 1,500 ms. Spikes of excitatory Pyr neurons and the three subtypes of inhibitory interneurons are illustrated by black and gray dots, respectively. For this plot, visual stimuli were given from 0 ms. (TIF) [file pcbi.1013306.s001.tif]

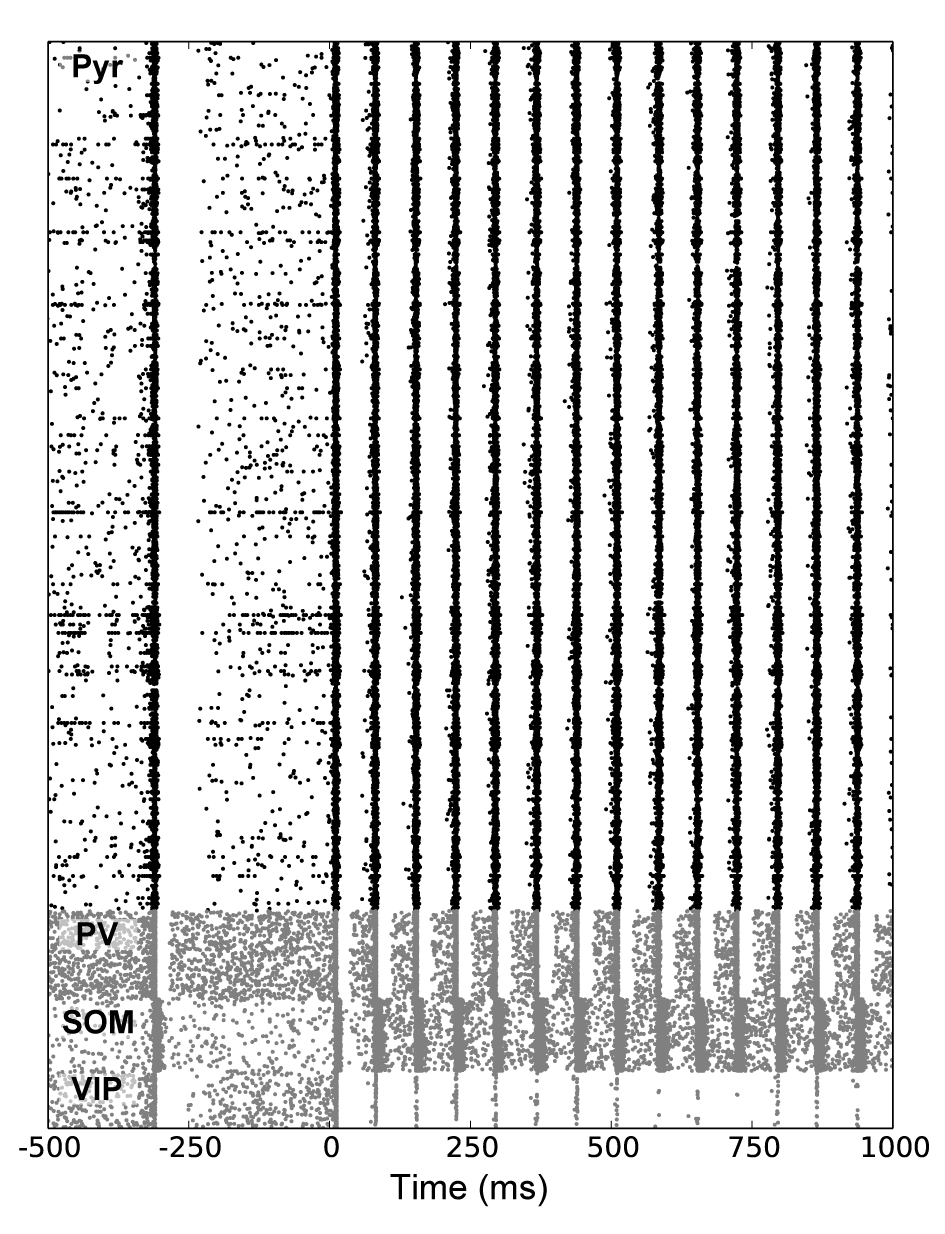

Supplement: S2 Fig — Conventions are same as those in Fig 2A in the main text and S1 Fig. (TIF) [file pcbi.1013306.s002.tif]

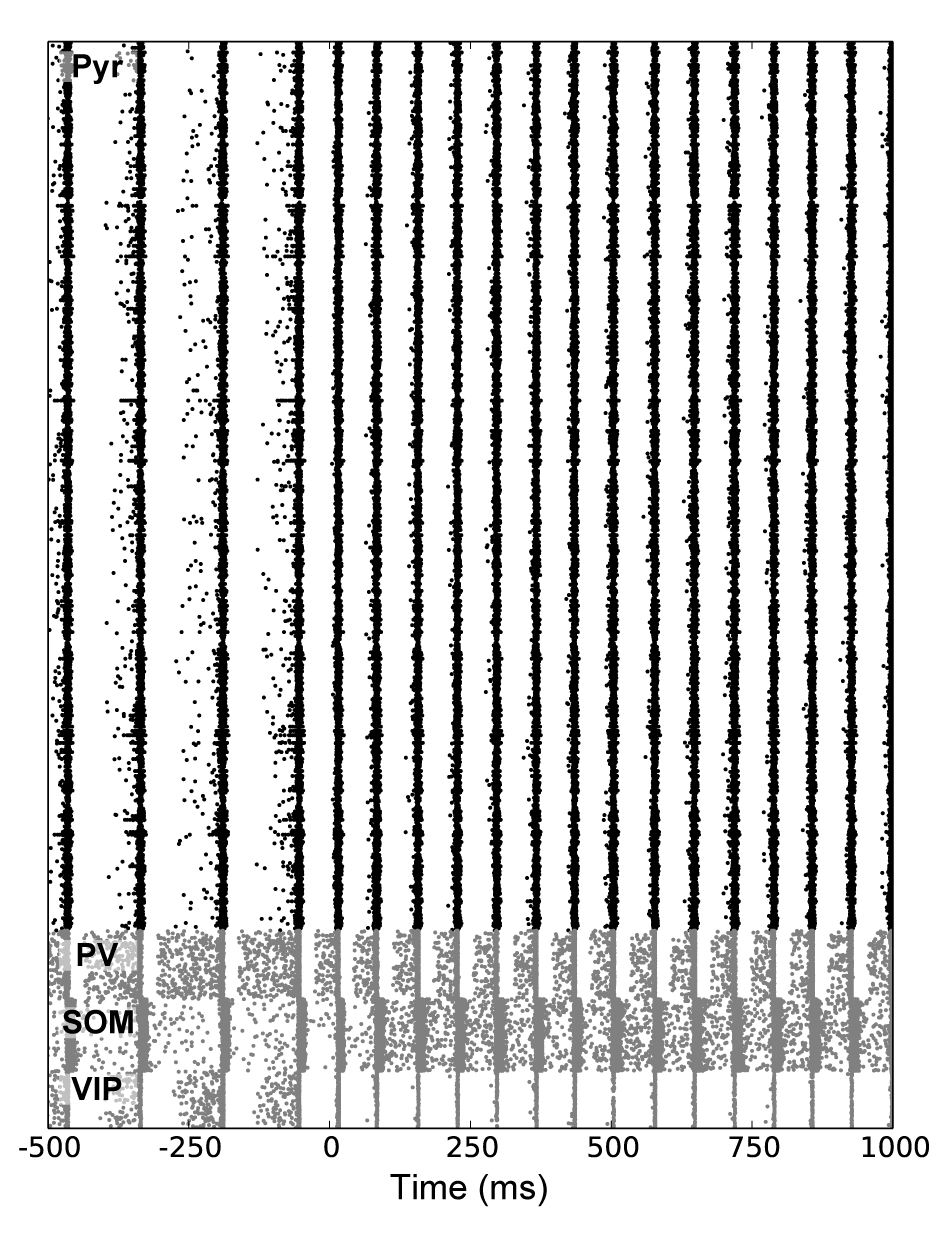

Supplement: S3 Fig — Conventions are same as those in Fig 2A in the main text and S1 Fig. (TIF) [file pcbi.1013306.s003.tif]

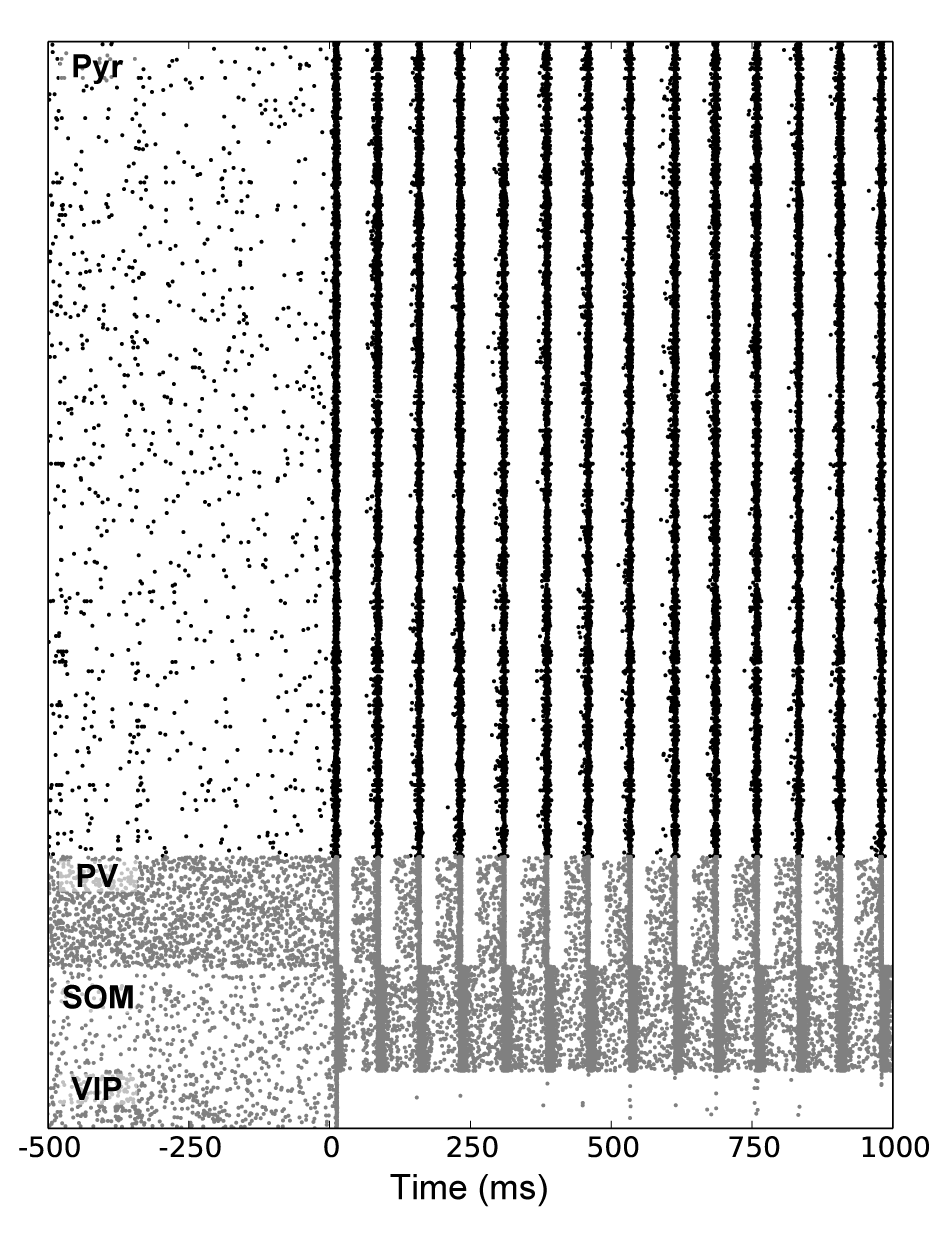

Supplement: S4 Fig — As shown in Fig 2A in the main text, raster plots showing all spike trains of the Pyr, PV, SOM, and VIP neuron populations for 1,500 ms. Conventions are same as those in Fig 2A in the main text. (TIF) [file pcbi.1013306.s004.tif]

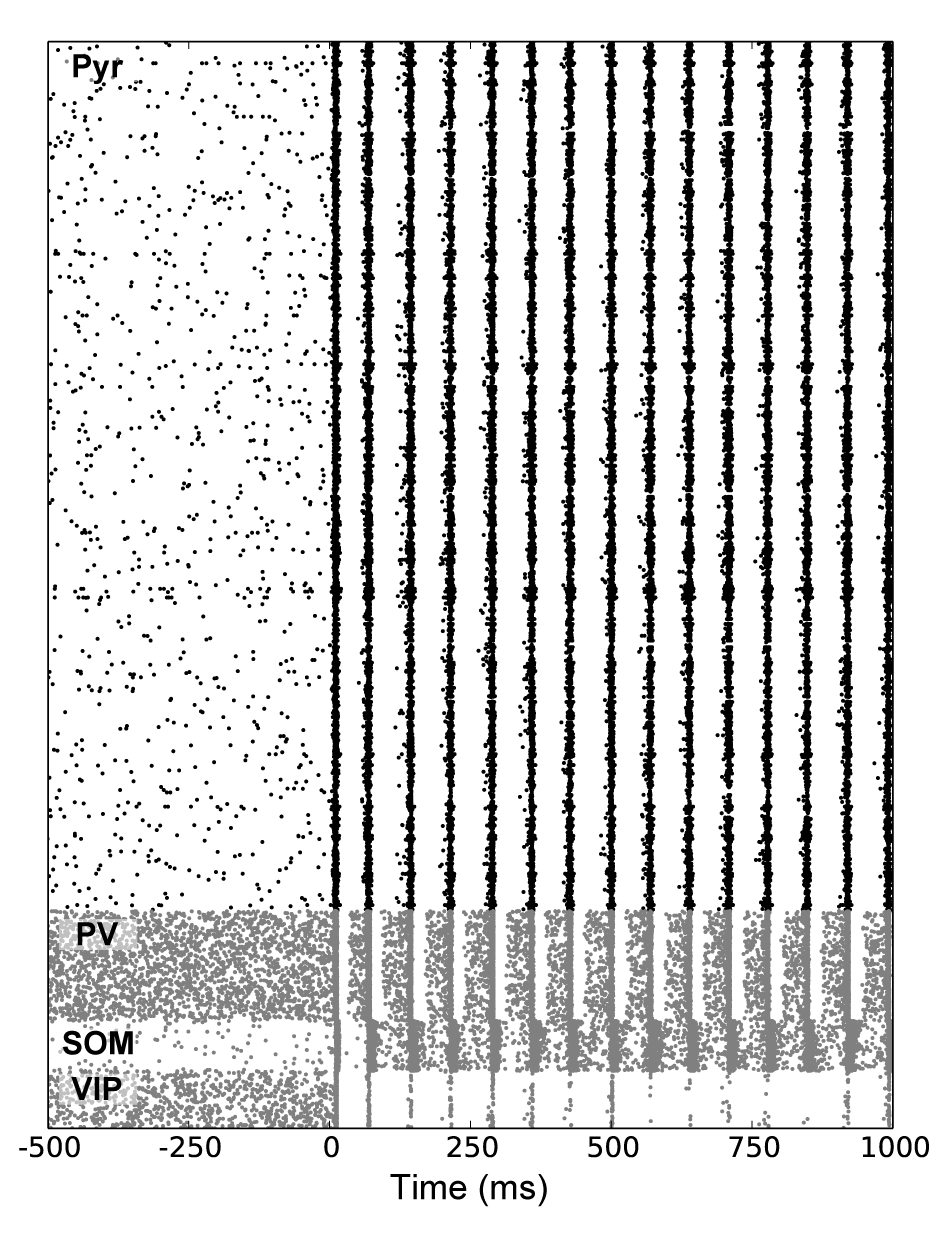

Supplement: S5 Fig — Conventions are same as those in Fig 2A in the main text. (TIF) [file pcbi.1013306.s005.tif]

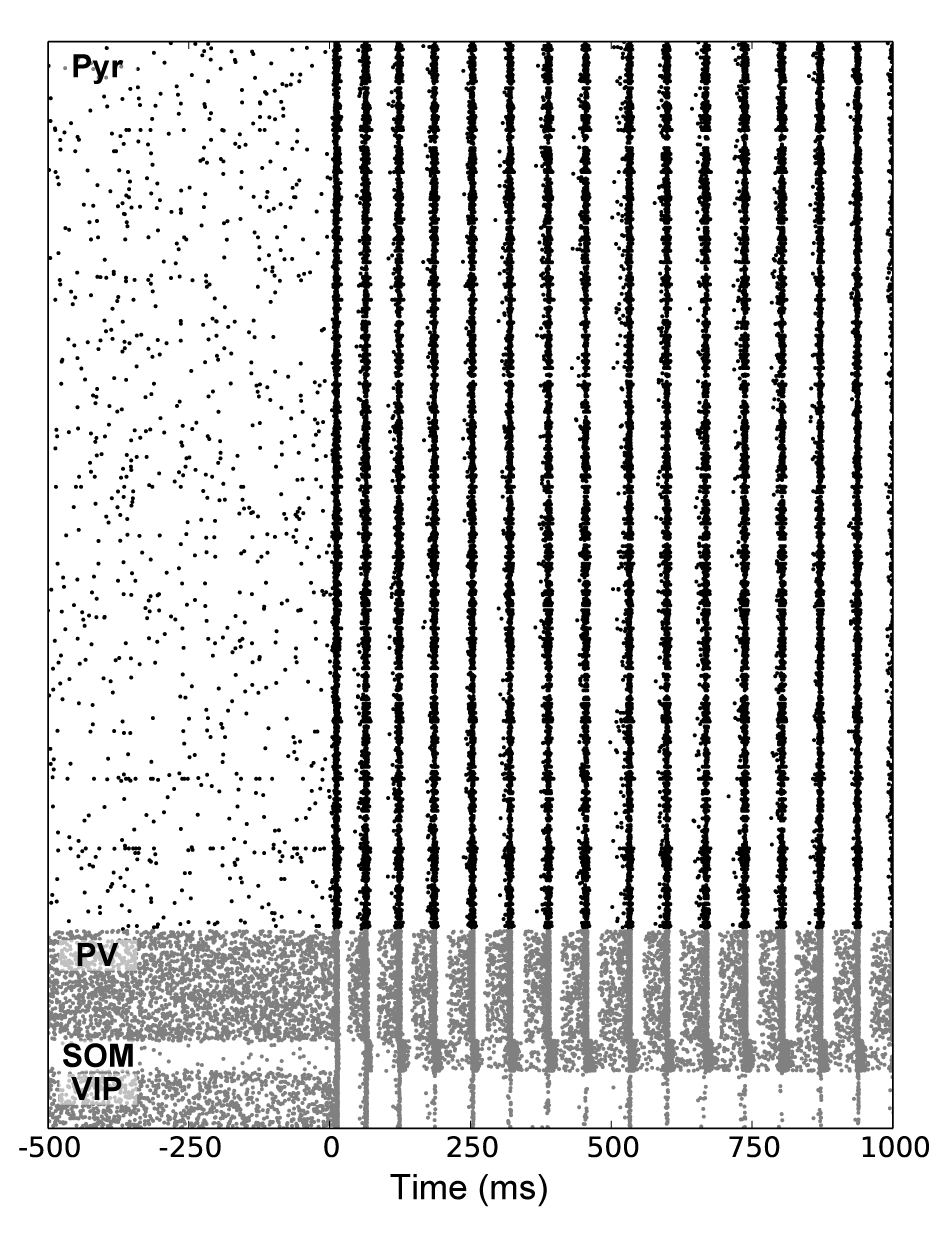

Supplement: S6 Fig — Conventions are same as those in Fig 2A in the main text. (TIF) [file pcbi.1013306.s006.tif]

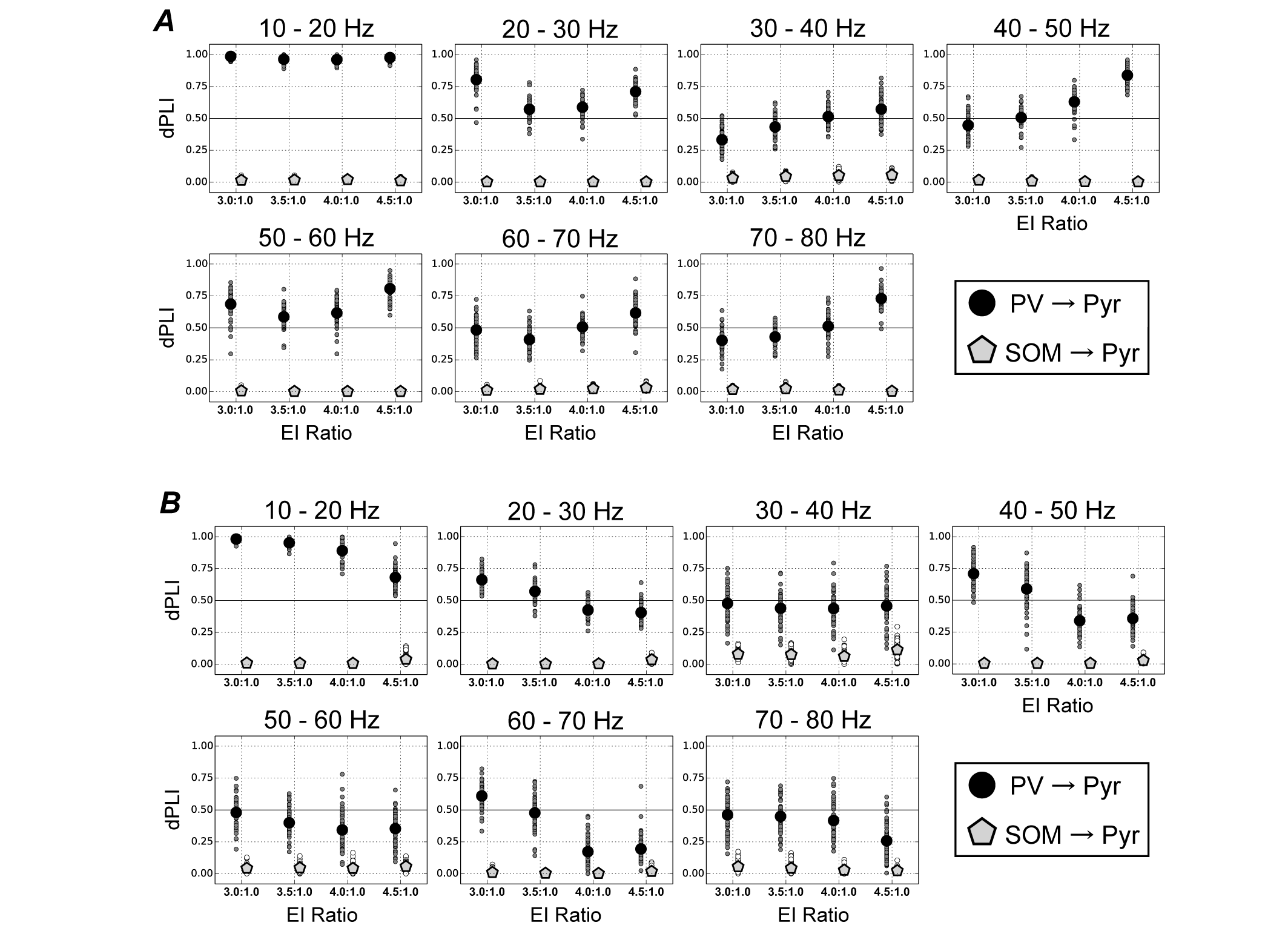

Supplement: S7 Fig — Conventions are same as those in Fig 5 in the main text. A. Values of dPLI from PV or SOM inhibitory interneurons to Pyr neurons as a function of E/I ratio with respect to Pyr and PV populations. B. Values of dPLI from PV and SOM to Pyr populations as a function of E/I ratios with respect to Pyr and SOM populations. The modulation patterns of dPLI values associated with the PSTH power peaks in the 10–20 Hz, 20–30 Hz, and 40–50 Hz frequency bands (Figs 3 and 4 in the main text) resemble those observed in other frequency ranges. (TIF) [file pcbi.1013306.s007.tif]

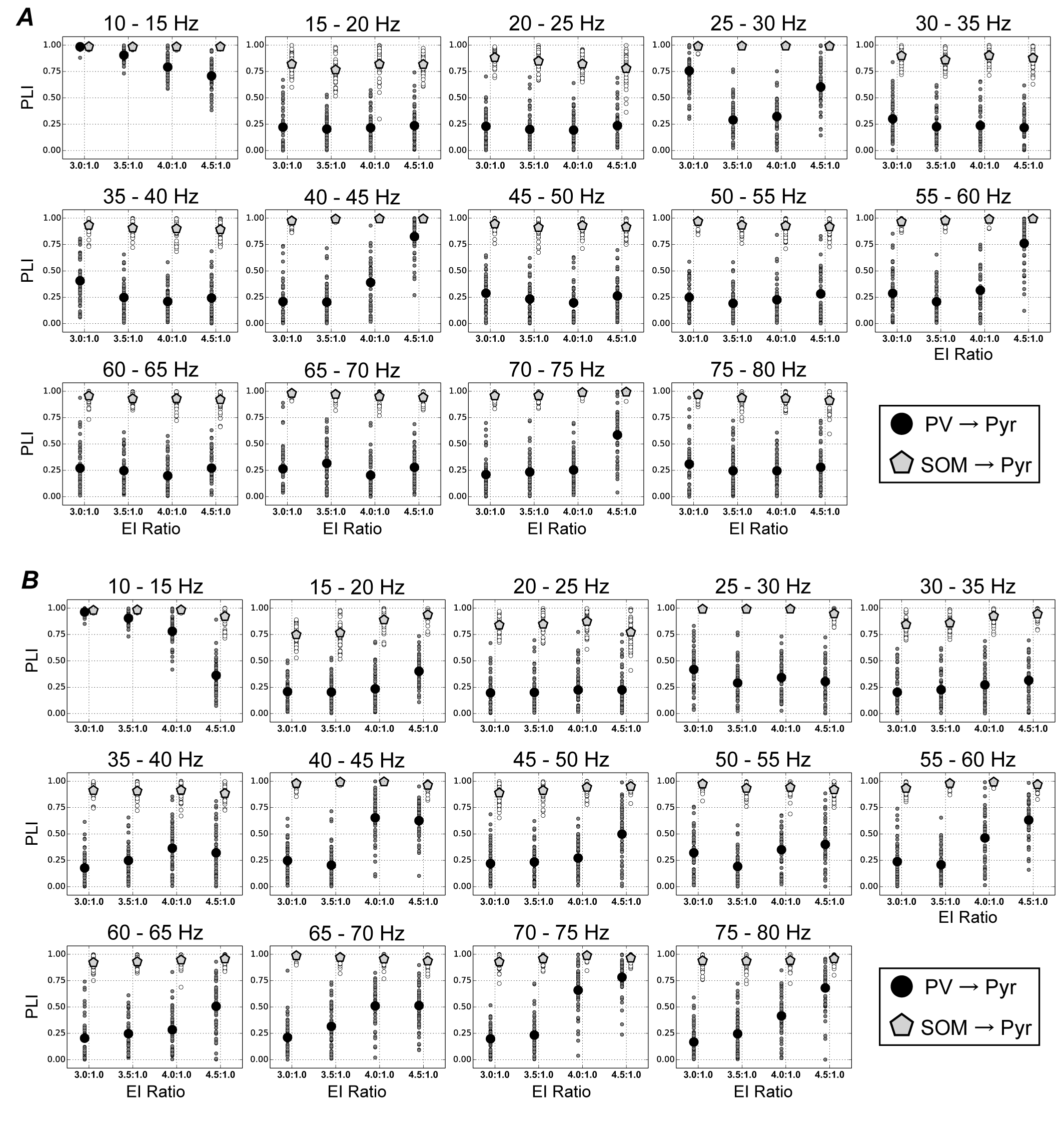

Supplement: S8 Fig — Black circles and gray pentagons represent the mean PLI values from PV to Pyr and SOM to Pyr populations, respectively. PLI values from PV to Pyr and from SOM to Pyr populations for each trial are shown by small black and gray circles, respectively. A. PLI values from PV or SOM inhibitory interneurons to Pyr neurons as a function of the E/I ratio for Pyr and PV populations. The PLI values between the SOM and Pyr populations (gray pentagons) were almost constant, irrespective of the frequency band and E/I ratio. By contrast, when the E/I ratio was increased from 3.5:1.0, the PLI values between the Pyr and PV populations (black circles) tended to exceed 0.25 at peak frequencies around 10–15 Hz, 25–30 Hz, and 40–45 Hz (see also Fig 3B). B. PLI values from PV and SOM to Pyr populations as a function of the E/I ratio for Pyr and SOM populations. As shown by the E/I ratio for the PV population (panel A), the PLIs from the SOM to Pyr populations (gray pentagons) did not fluctuate around values of one. Additionally, when the E/I ratio exceeded 3.5:1, the PLI values between the Pyr and PV populations (black circles) tended to increase as the frequency increased. Furthermore, the frequencies of the peaks in the PSTH power around 10–15 Hz and 40–45 Hz (Fig 4B) appeared to correspond to strong PLI values, at least in part. (TIF) [file pcbi.1013306.s008.tif]
